# Diverse novel RNA viruses in the cryosphere of the Tibetan Plateau

**DOI:** 10.1093/nsr/nwaf361

**Published:** 2025-08-28

**Authors:** Yongqin Liu, Zhihao Zhang, Nianzhi Jiao, Yong-Guan Zhu, Rui Zhang, Guillermo Dominguez-Huerta, Haina Wang, Meiling Feng, Rong Wen, Mukan Ji, Qiang Zheng, Pengfei Liu, Tandong Yao

**Affiliations:** Center for Pan-third Pole Environment, Lanzhou University, China; State Key Laboratory of Tibetan Plateau Earth System, Resources and Environment (TPESRE), Institute of Tibetan Plateau Research, Chinese Academy of Sciences, China; Chayu Monsoon Corridor Observation and Research Station for Multi-Sphere Changes, Xizang Autonomous Region, China; University of Chinese Academy of Sciences, China; Center for Pan-third Pole Environment, Lanzhou University, China; State Key Laboratory of Tibetan Plateau Earth System, Resources and Environment (TPESRE), Institute of Tibetan Plateau Research, Chinese Academy of Sciences, China; University of Chinese Academy of Sciences, China; Innovation Research Center for Carbon Neutralization, Fujian Key Laboratory of Marine Carbon Sequestration, Xiamen University, China; College of Ocean and Earth Sciences and State Key Laboratory of Marine Environmental Science, Xiamen University, China; Research Center for Eco-Environmental Sciences, Chinese Academy of Sciences, China; Institute for Advanced Study, Shenzhen University, China; Centro Oceanografico de Ma laga, Instituto Español de Oceanografía, IEO-CSIC, Spain; Key Laboratory of Groundwater Circulation and Environmental Evolution (China University of Geosciences, Beijing), Ministry of Education, China; Department of Biological Sciences, University of Bergen, Norway; Center for Pan-third Pole Environment, Lanzhou University, China; Key Laboratory of Pan-third Pole Biogeochemical Cycling, China; Center for Pan-third Pole Environment, Lanzhou University, China; Key Laboratory of Pan-third Pole Biogeochemical Cycling, China; Center for Pan-third Pole Environment, Lanzhou University, China; Key Laboratory of Pan-third Pole Biogeochemical Cycling, China; Innovation Research Center for Carbon Neutralization, Fujian Key Laboratory of Marine Carbon Sequestration, Xiamen University, China; College of Ocean and Earth Sciences and State Key Laboratory of Marine Environmental Science, Xiamen University, China; Center for Pan-third Pole Environment, Lanzhou University, China; Key Laboratory of Pan-third Pole Biogeochemical Cycling, China; State Key Laboratory of Tibetan Plateau Earth System, Resources and Environment (TPESRE), Institute of Tibetan Plateau Research, Chinese Academy of Sciences, China

**Keywords:** RNA virus, RNA-dependent RNA polymerase (RdRp), bacteriophage, auxiliary metabolic genes (AMGs), the Tibetan Plateau cryosphere

RNA viruses are a type of viruses that use ribonucleic acid (RNA) as their genetic material. The diversity of RNA viruses is rapidly increasing [1,2], with over 10^5^ species of RNA viruses being assigned to over 180 phylum- or class-level superclades, with their hosts spanning bacteria, fungi, plants and animals [2]. Environmental RNA viruses play important roles in global biogeochemical cycling and ecosystem function maintenance beyond their impacts on public health. Therefore, understanding the biogeography and genetic characteristics of environmental RNA viruses can provide novel insights into the dynamics and the ecological roles in natural ecosystems [3].

The Tibetan Plateau (TP) possesses the largest area of cryosphere in the mid–low latitude region. Microbes are the main forms of life that have adapted to harsh conditions like intense UV radiation, extreme temperature fluctuations and low atmospheric pressure [4] of the TP cryosphere (TPC). Therefore, we hypothesize that the diversity of RNA viruses in the TPC would be distinct from those in other environments due to the unique environmental conditions, and this unique RNA virus community may play a crucial role in shaping microbial dynamics and influencing biogeochemical processes in the TPC ecosystems. However, except for our recent work on one alkaline saline lake of the TPC [5], the RNA virus communities of the TPC environment have rarely been explored. Additionally, the TP is highly sensitive to climate change [6]. Therefore, the RNA viruses stored in the TPC will be released into downstream ecosystems, substantially influence the microbial community and have an impact on public health. Here, to explore the diversity, ecological functions and potential pathogenic risks of TPC RNA viruses, we performed metatranscriptome sequencing of 79 samples, covering glacier [28 samples, including snow (5), ice (5) and cryoconites (18)], proglacial lake (6), upland (16) and wetland (29) in permafrost regions from the TPC (Fig. 1a and b; Table S1).

**Figure 1.**
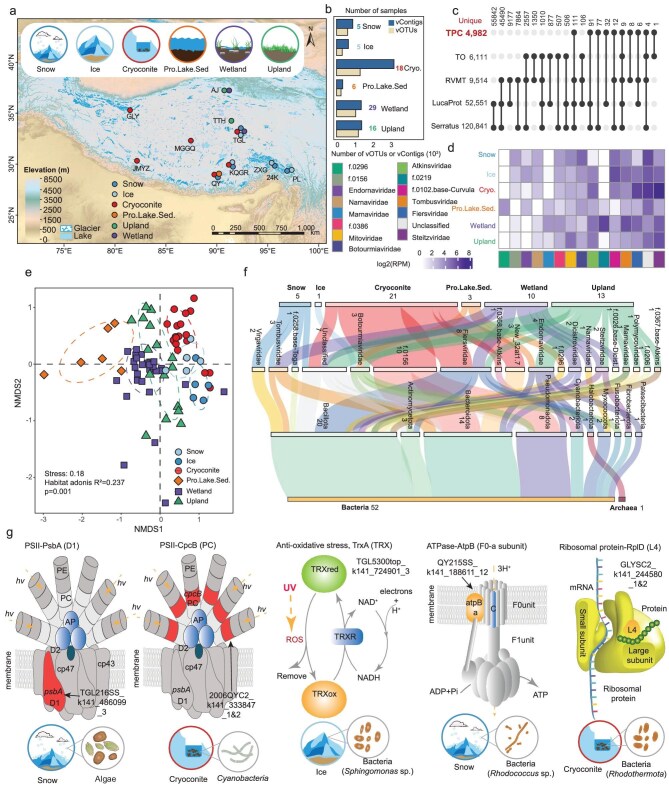
Characterization of the TPC RNA virome. (a) Overview of metatranscriptome sampling sites across the Tibetan Plateau. (b) Sample sources and the number of vContigs and vOTUs obtained from each habitat. (c) Upset plot showing the number of unique TPC vOTUs compared to the *Tara* Oceans, the RVMT, the Serratus and the LucaProt datasets. (d) Average relative abundance of RNA viral families across different habitats. The legend for (d) is shown on its left. (e) Non-metric multidimensional scaling (NMDS) plot showing the clustering of RNA virus communities. (f) Sankey plot showing the linkage of vContigs to their potential prokaryotic hosts. The number beneath each node indicates the number of vContigs assigned to the corresponding viral group. (g) Cartoon showing the cellular location and function of selected AMGs from snow, ice and cryoconite, and the source and potential host of vContigs with the corresponding AMGs. Pro.Lake.Sed., proglacial lake sediments; Cryo., cryoconite. Review drawing number: GS 京 (2025) 2049号.

We identified RNA viruses through mining RNA-dependent RNA polymerase (RdRp), the single hallmark protein that is shared by all orthornavirans [7] and required for replication. We also inferred that the RNA viruses encoded auxiliary metabolic genes (AMGs) through functional annotation, and assessed their pathogenic risks through host assignments and genome feature-based prediction.

***RNA virus community composition of the TPC.*** We obtained 8799 RNA viral contigs (vContigs) (Fig. 1b; Table S2), of which 6893 vContigs could be assigned to 6 viral phyla and 128 families according to the International Committee on Taxonomy of Viruses (ICTV) and RNA Viruses in Metatranscriptomes (RVMT) framework [2] (Figs S1 and S2; Table S3). The 8799 vContigs were clustered into 5333 species-level virus

operational taxonomic units (vOTUs; Fig. 1b; Table S3), with only 351 being shared with viral genomes from the global RNA virome datasets and 4982 vOTUs (93.4%) being TPC unique (Fig. 1c). Therefore, our work substantially expands the limits of the known RNA virosphere.

The composition of TPC RNA viruses varied across habitats (Fig. 1d; Figs S3 and S4). Prokaryotic RNA virus families dominate most environments, with *Steitzviridae, Fiersviridae* and *Atkinsviridae* as the most abundant groups (Fig. 1d; Fig. S4B). Other groups that were abundant in at least a subset of samples from a certain habitat mainly included *Tombusviridae, Botourmiaviridae, Mitoviridae, Marnaviridae, Narnaviridae* and *Nodaviridae* (Fig. 1d; Fig. S4C). The difference of habitats is most likely the key factors that shape the RNA virus community variations within the TPC. See Supplementary Information in the Supplementary  data for more discussions.

***Diversity of the TPC RNA virus communities.*** The observed viral richness was between 21 and 483 (median ranged from 97 in proglacial lake sediment to 219 in snow) and the Shannon's H was between 1.50 and 6.9 (median ranged from 3.6 in cryoconite to 5.8 in snow). The richness of the RNA virus communities of the glacier habitats was slightly higher than other habitats while the Shannon's H was similar among all habitats (Fig. S5). Comparison with the global RNA virome revealed a high diversity of TPC RNA viruses (see Supplementary  Information).

The TPC RNA virus communities showed remarkable habitat specificity (PERMANOVA, Habitat *R^2^* = 0.237; Fig. 1e). The specificity of the TPC RNA virus communities was validated by the pairwise PERMANOVA analysis (all pairwise *P* < 0.001; Fig. S6). In addition, we found that 77 (60.2% of 128) RNA viral families were enriched in certain habitats (Fig. S7), which further supports the strong habitat specificity of TPC RNA virus communities (see Supplementary  Information).

***Expansion of the prokaryotic RNA viruses and their hosts.*** For the vOTUs with a family-rank taxonomic assignment, 3762 could be linked to the putative hosts, including prokaryotes (2175) and eukaryotes (1587) (Figs S8 and S9; Tables S3–S5). Among all the 68 established RNA viral families with host assignments, 14 and 54 of them were predicted to infect prokaryotes and eukaryotes, respectively (Figs S8 and S9). The TPC RNA virus communities were dominated by the prokaryotic RNA viruses in ice, cryoconite, wetland and upland (Fig. S4B; Fig. S9C and D), in contrast to the global RNA virome dataset (e.g. RVMT [2], LucaProt and *Tara* Oceans datasets [8, 9]), which are dominated by eukaryotic RNA viruses. Most likely the harsh environmental conditions of TPC mainly sustains prokaryotes, which support a higher proportion of prokaryotic RNA viruses (see Supplementary  Information).

We detected linkages between 46 TPC vContigs and 36 prokaryotic metagenome-assembled genomes (MAGs) affiliated with 10 phyla (Fig. 1f; Fig. S10; Tables S5 and S6). For these RNA vContigs, 39 are previously known as bacterial (*Fiersviridae* and *Steitzviridae*) and eukaryotic (*Mitoviridae, Botourmiaviridae*, family *f.0156* and *Endornaviridae*) viruses according to ICTV and RVMT, respectively (see Supplementary  Information for more discussions). In addition, we revealed the linkage between seven unclassified vContigs with bacterial MAGs affiliated with Bacteroidota, Pseudomonadota (formerly Proteobacteria), Bacillota (formerly Firmicutes) and Actinomycetota (Fig. 1f; Fig. S10; Table S6). Moreover, we observed one vContig (*Fiersviridae*) linked to one archaeal MAG (*Halobellus* sp. of Halobacteriota) (Fig. 1f; Table S6). In summary, our results provided additional evidence on RNA virus and prokaryote interactions, supporting that the diversity of prokaryotic RNA viruses is still largely unexplored and their roles in mediating biogeochemical cycling are underestimated [3] (see Supplementary  Information).

***Eukaryotic RNA viruses and their potential risks.*** There were 54 RNA viral families associated with eukaryotes, which were dominated by *Tombusviridae, f.0219* and *Botourmiaviridae* (Fig. 1d; Fig. S4C). Potential RNA viral pathogens were detected. Potential plant-infecting viruses with relatively high reads per million (RPM) included *Botourmiaviridae* in snow (median RPM 141.0), *Tombusviridae* in wetland (272.6) and *Endornaviridae* in ice (21.9; Fig. 1d; Fig. S4C). In addition, viruses that might infect animals were also detected across habitats, with some showing high abundance in specific environments (Fig. S4C). For example, maximum RPMs exceeded 11 693 for *Nodaviridae* and 752 for *Flaviviridae* in wetlands. Other families, including f.0296, *f.0292* and *Hepeviridae*, also showed RPMs of >20, while the rest (e.g. *Astroviridae*) remained low across all samples (max RPM < 10; see Supplementary Information).

In order to further identify potential zoonotic RNA viruses in the TPC, we applied Zoonotic_rank, a machine-learning and genome feature-based approach in addition to the putative host assignment. This analysis showed that only 0.4% (Table S7) of the 8799 vContigs were categorized as zoonotic viruses with a rank of ‘very high’, indicating low public health risks (see Supplementary  Information).

***AMGs encoded by TPC RNA viruses.*** We identified 39 AMGs from 15 vContigs out of the 8799 vContigs. These AMGs mainly belonged to the functional categories including translation (18), carbohydrate metabolism (4), transportation (5), energy metabolism (5), chaperones (1) and peptidases (1) (Fig. S11; Tables S8 and S9). Thirty-five (89.7% of 39) were first reported here (Table S8). In the transcriptome, the abundance of most AMGs (Fig. S12) was markedly lower than RdRp-coding genes. However, the abundance of five AMGs from glacier environments was profoundly higher than that of RdRp genes, including *psbA* (encodes photosystem II core reaction center protein D1, 17.3-fold higher), *cpcB* (phycocyanin protein beta chain, 54.9 and 34.3, respectively), *atpB* (ATPase F0a protein, 3.1), *trxA* (thioredoxin protein, 5.9) and *rplD* (large subunit ribosomal protein L4, 5.1) (Fig. 1g; Fig. S12B).

TrxA, ATPase and ribosomal proteins play crucial roles in removing reactive oxygen species (ROS) indirectly induced by high levels of UV radiation to avoid cellular damage, or enhance the metabolism, and are resistant to the low temperature and nutrient conditions on the glacier. Most excitingly, we found that RNA viruses from the glacier encode two key proteins (PsbA and CpcB) of the core photosynthesis system. *psbA* has been detected in marine cyanophage, which helps the host cope with radiation damage [10]. In our dataset, the RNA virus with the *psbA* gene was identified from snow and was predicted to infect eukaryotic algae, and *cpcB* was identified in cyanobacteria-infecting RNA virus from cryoconite (Fig. 1g; Fig. S13). Thus, the *psbA* and *cpcB* genes may have similar functions as those encoded in cyanophage [10], which is to cope with radiation-caused photosynthesis-related protein damage. Thus, we propose that on the glacier surface, the RNA viruses with these five AMGs would facilitate the adaptation and proliferation of their hosts, which may influence the melting of glaciers by reducing the albedo of the glacier surface.

In conclusion, we obtained the first RNA viral genome dataset recovered from metatranscriptomes of the TPC, revealing diverse novel RNA viruses and their hosts, which fills a unique geographic and ecosystem gap of the global RNA virome atlas. The TPC RNA virus communities exhibited a remarkable habitat specificity. In particular, the TPC RNA virus communities are dominated by viruses infecting prokaryotes, distinct from those reported previously and highlighting an overlooked role of RNA viruses in mediating biogeochemical cycling in the cryosphere. Our work provides a wealth of resources for global RNA virosphere study from the cryosphere and greatly expands our understanding of the diversity and ecological roles of environmental RNA viruses.

## Supplementary Material

nwaf361_Supplemental_File
