# Supplementary material for: Diverse novel RNA viruses in the cryosphere of the Tibetan Plateau
Source: Natl Sci Rev. 2025 Aug 28;12(11):nwaf361. doi: 10.1093/nsr/nwaf361 (PMC12581897; doi:10.1093/nsr/nwaf361)
Supplement: nwaf361_Supplemental_File [file nwaf361_supplemental_file.docx]

**Supplementary Information for**

**Diverse novel RNA viruses in the cryosphere of the Tibetan Plateau**

Yongqin Liu, Zhihao Zhang, Nianzhi Jiao, Yongguan Zhu, Rui Zhang, Guillermo Dominguez-Huerta, Haina Wang, Meiling Feng, Rong Wen, Mukan Ji, Qiang Zheng, Pengfei Liu, Tandong Yao

*Pengfei Liu

Email: liupf@lzu.edu.cn

**This PDF file includes:**

Supplementary Results and Discussions

Figures S1 to S21

Titles for Supplementary Tables S1 to S12

SI References

**Other supporting materials for this manuscript include the following:**

TPC RNA virus genome datasets, including the RdRps amino acid sequences, vContigs sequences, full functional annotations of the 8,799 RNA virus genomes; Supplementary Tables S1-S12; Supplementary Figures S1-S21; alignment files for AMGs domain annotation related to Fig. S17 and S18 are available at figshare (<https://doi.org/10.6084/m9.figshare.c.7390684>).

In addition, the relative abundance of RNA viruses expressed in the form of Transcripts Per Million mapped reads (TPM) are also available at figshare (<https://doi.org/10.6084/m9.figshare.c.7390684>).

Supporting Information Text

Supplementary Results and Discussions

### Comparison of RNA virus communities between habitats

Our findings demonstrate that RNA virus communities exhibit strong habitat specificity (Fig. 1D&E; Figs. S3-S4), with the RPM of RNA viral families varying significantly across different habitats (Fig. S4). Prokaryotic RNA viruses dominate the Tibetan Plateau Cryosphere (TPC) RNA virome, particularly in cryoconite, wetland and upland (Fig. S4B). The most abundant prokaryotic RNA viral families include *Steitzviridae*, *Atkinsviridae*, *Fiersviridae*, and f.0368.base-Atkins, with median RPM of 1,312.1 (cryoconite), 134.1 (cryoconite), 406.8 (cryoconite), and 14.0 (ice), respectively (Fig. S4B). Other notable prokaryotic RNA viral families with relatively low abundances include f.0144 (8.7, proglacial lake sediment), *Blumeviridae* (8.8, cryoconite), f.0367.base-Atkins (9.8, upland), *Solspiviridae* (3.0, upland), *Cystoviridae* (2.7, snow) and f.0153 (1.7, proglacial lake sediment). Minor groups, such as *Duinviridae*, had median RPM value <1 (Fig. S4B).

In addition to prokaryotic RNA viruses, eukaryotic RNA viral families also showed significant dominance in certain habitats, particularly in ice, snow, and proglacial lake sediments (Fig. S4C). For example, in ice, *Tombusviridae* (median RPM, 121.1), *Botourmiaviridae* (28.8) and *Endornaviridae* (22.0) were the top three most abundant families. In snow, the dominant families included *Botourmiaviridae* (141.0), *Tombusviridae* (44.6), and *Mitoviridae* (43.5). In proglacial lake sediments, f.0296 (31.1), *Nodaviridae* (23.8), *Botourmiaviridae* (23.0), *Tombusviridae* (10.6), and f.0292 (10.1) were the top five most abundant families. Wetlands were dominated by *Tombusviridae* (272.6), *Marnaviridae* (29.3) and *Mitoviridae* (9.6). In uplands, the top families included *Mitoviridae* (91.7), *Tombusviridae* (18.8) and *Botourmiaviridae* (15.4). Unclassified RNA viral families also constituted a substantial proportion of viral communities, particularly in glacier ecosystems: cryoconites (median RPM, 647.2), ice (214.5), and snow (339.4) (Fig. 1D; Fig. S4). Furthermore, 77 (60.2%) of the 128 RNA viral families were enriched in specific habitats, with 2, 3, 19, 12, 18, and 23 families enriched in snow, ice, cryoconite, proglacial lake sediment, wetland, and upland, respectively (Fig. S7). These findings further underscore the habitat specificity of the TPC RNA virome.

We propose that environmental conditions and biomes are key drivers of the observed differences in RNA virus communities across habitats. Glaciers, characterized by intense UV radiation, low temperatures, and low nutrient concentrations, represent extreme environments that nonetheless support diverse microbial communities, including bacteria, algae, fungi, and microfauna such as rotifers and tardigrades[1]. The glacial surface encompasses distinct habitats such as snow, ice, and cryoconite, with cryoconite serving as metabolic hotspots for biogeochemical cycling [2]. Microbial communities in these habitats differ significantly; for example, cyanobacteria dominate carbon fixation in cryoconite, while eukaryotic *Zygnematales* and *Chlamydomonadales* are prevalent on ice surfaces and in snow, respectively[3]. Proglacial lakes, fed by glacial meltwater, are cold and oligotrophic environments with microbial communities primarily composed of bacteria, alongside phytoplankton and zooplankton[4-6]. Glacial melting significantly influences these microbial communities, contributing to their composition [5].

Similarly, the wetlands and uplands of the TP are also extreme environments marked by temperature fluctuations, UV radiation, and seasonal freeze-thaw cycles, with microbial communities dominated by bacteria, archaea, and fungi, as well as algae, protozoa, and insects[7-13]. Due to differences in moisture and vegetation, the biological composition of wetland and upland also differs significantly[13].

Current understanding of the factors shaping environmental RNA virus communities remains limited [14-18]. In marine ecosystems, environmental parameters such as temperature and nutrient availability are key drivers [19]. In terrestrial ecosystems, factors including organic content [14], water content[18], plant presence[15], type of cultivar[15], soil depths[20] and soil types[18]. In addition, the types and abundances of eukaryotes play a critical role in both marine and terrestrial ecosystems [14, 15]. Chen et al. further demonstrated that environmental factors have a greater impact on the abundance and diversity of plant, fungal, and bacterial viruses than on animal viruses [14]. Collectively, the pronounced differences in RNA virus communities across habitats (Fig. 1D&E; Figs. S3-4, S7) and the findings of these studies support the hypothesis that environmental conditions and biomes are primary determinants of RNA virus community specificity (see the Main Text).

### Shared vOTUs across different habitats

In addition to the RNA virus diversity and community structure, we further investigated the common RNA viral groups that shared across various habitats of the TPC (Fig. S14). The majority of vOTUs (4,898; 91.8% of 5,333) were habitat-specific, while a smaller proportion (435; 8.2%) were shared among multiple habitats. The largest group of shared vOTUs are from glacier ecosystems, with 336 vOTUs shared among snow, ice, and/or cryoconite, accounting for 77.2% of all shared vOTUs (Fig. S14A). The second most shared vOTUs were between wetland and upland (75; 17.2%) (Fig. S14A)

The high degree of virus sharing among snow, ice, and cryoconite is likely due to their hydrological connectivity via meltwater [2] and their relatively similar environmental conditions. In contrast, wetland and upland environments were more similar with each other than with other habitats. For example, during the non-glacial melt season, the water table in the Tanggula wetland decreases [21], leading to physicochemical conditions more akin to those of upland, which may explain their higher proportion of shared viruses. Additionally, the small number of shared vOTUs between glacier habitats and proglacial lake sediments likely reflects the influence of glacier meltwater on microbial communities in these lakes [22-24]. These findings suggest that glacier RNA viruses may enter proglacial lakes via meltwater and persist in sediments, highlighting the potential impacts of glacier melting on downstream ecosystems.

Although shared vOTUs represented a small proportion of total vOTUs, they showed a relatively high RPM in TPC RNA virus communities, particularly in snow, ice, cryoconite and wetland (Fig. S14B). The shared vOTUs were dominated by four established RNA viral families with median RPM over 1,000, including *Steitzviridae* (707.2, cryoconite), f.0219 (415.0, wetland), f.0102.base-Curvula (290.8, ice), *Tombusviridae* (151.4, wetland) (Fig. S14B).

### Variations of RNA virus compositions within the same type of habitat

In addition, we observed significant variations in RNA virus compositions among samples from the same habitat type (Figs. S3, S4&S9). For example, some families (e.g., *Tombusviridae*) that are dominant in some samples but may be absent or poorly represented in other samples from the same habitat type. These variations can be attributed to geographic, climatic, and temporal factors.

Geographically, glaciers on the TP are influenced by distinct atmospheric circulations. Glaciers in the south and southeast are primarily affected by Monsoon, while those in the north and northwest are influenced by the Westerlies [25]. Additionally, radiation levels are lower in the southeast compared to the north and northwest [26]. For upland, samples were collected from two geographically distant regions (TTH and AJ; Fig. 1A; Table S1) with differing altitudes and annual mean air temperatures, resulting in variations in soil physicochemical properties.

Temporally, proglacial lake sediment samples were collected in January and May (Table S1), representing the non-melting and early melting seasons, respectively. These periods differ markedly in temperature and the influence of glacier meltwater [27]. Wetland samples were collected in August, April, and June, corresponding to the growing, frozen, and freeze-thaw cycling season, respectively [21]. Given the dynamic nature of RNA virus communities and the broad geographic and temporal scope of our sampling, the observed variations within the same habitat type are not unexpected. These findings highlight the importance of considering spatial and temporal heterogeneity when studying RNA virus ecology in extreme environments.

### Comparison of the RNA virome between the TPC and other cryosphere environments

Environmental RNA viruses in the global cryosphere remain undersampled and understudied, with only a limited number of studies focusing on RNA virus communities in these ecosystems [17, 20, 28, 29]. When compared to our findings, these studies reveal a substantial overlap in RNA virus families (Fig. S15), but significant differences in viral community composition.

For example, Wu et al.[17] investigated RNA viruses in thawed permafrost soils from the Arctic (Alaska), where double-stranded RNA viruses such as *Reoviridae* and *Hypoviridae*, as well as single-stranded RNA viruses like *Rhabdoviridae* (negative-sense) and *Leviviridae* (positive-sense), dominated the communities. Wen et al. [20] examined RNA viruses along a permafrost soil profile in the TPC, using cores up to 100 meters deep. They observed depth-dependent variations, with prokaryotic viruses (*Leviviridae*, *Steitzviridae*), plant viruses (*Botourmiaviridae*, *Bromoviridae*), fungal viruses (*Narnaviridae*, *Totiviridae*), and vertebrate viruses (*Paramyxoviridae*) being the most abundant. In contrast, Wu et al. [28] explored RNA viruses in the second-largest lake on the TP, finding that lake water communities were dominated by f.0292, f.0102.base-Curvula, *Mitoviridae*, *Benyviridae*, f.0296, and *Narnaviridae*.

The observed differences in RNA virus communities likely stem from multiple factors, including geographic location (e.g., the TP vs. Arctic), sample processing methods (e.g., *in-situ* vs. post-incubation), and significant environmental variations across habitats, as discussed earlier. Additionally, methodological differences in RNA virus analysis may contribute to these discrepancies. For example, Wen et al. [20] relied on nucleotide sequences similarity comparisons of assembled contigs for RNA virus identification, while other studies, including ours, employed RdRp-based mining approaches.

Despite these differences, the unique RNA virus communities observed across cryosphere ecosystems underscore their adaptation to extreme environments and highlight the need for more comprehensive studies of the global cryosphere RNA virome[30]. Such efforts will enhance our understanding of RNA virus diversity, ecology, and their roles in these critical ecosystems.

### Comparison of alpha diversity of TPC RNA viruses with the global RNA virome

Hou et al. reported significant variations in the Shannon index of RNA virus communities across global environments, with values ranging from 1 to 6[30]. High-diversity environments, such as leaf litter, wetlands, freshwater, and wastewater, exhibited Shannon indices averaging between 5 and 6, while low-diversity environments, including hot springs, air, and the deep biosphere, ranged from 0 to 1 [30]. The TP is known for its rich biodiversity, hosting diverse plants, animals, and microbial communities[31, 32]; however, the diversity of environmental RNA viruses in this region has not been previously explored.

Using the Shannon index, we assessed RNA virus diversity across the TPC habitats. The diversity of RNA viruses in TPC habitats ranged from 1.5 to 6.8 (Fig. S5), with snow exhibiting the highest value (approximately 7), comparable to that of leaf litter [30]. Other TPC habitats, such as wetlands, freshwater, and proglacial lake sediments, showed Shannon indices between 1.6 and 6.3, similar to other high-diversity global environments like wetlands, freshwater, and wastewater (~5) [30]. These findings demonstrate that, despite being an extreme environment, the TPC harbors a remarkably high diversity of RNA viruses, comparable to some of the most diverse ecosystems globally.

### Potential RNA viral pathogens

The RNA viral families identified in the TPC environment included potential pathogens of both plants and animals. We detected 14 plant RNA viral families: *Alphaflexiviridae* (vContigs =2), *Amalgaviridae* (6), *Aspiviridae* (14), *Benyviridae* (1), *Botourmiaviridae* (546), *Closteroviridae* (3), *Deltaflexiviridae* (4), *Endornaviridae* (53), *Potyviridae* (4), *Secoviridae* (6), *Solemoviridae* (47), *Tombusviridae* (228), *Tymoviridae* (4), and *Virgaviridae* (15) (Fig. S9; Table S4). Most of these families were also reported by Wen et al. in permafrost soils of TTH (Fig. S15; Table S10), along with four additional families: *Betaflexiviridae*, *Fimoviridae*, *Retroviridae*, and *Tumbusviridae*[20]. Several of these viruses could significantly impact agricultural productivity. For instance, *Alphaflexiviridae*, *Solemoviridae*, and *Potyviridae* are known pathogens of solanaceous crops such as potatoes, tomatoes, and peppers. In Arctic permafrost soils, Wu et al. identified *Reoviridae* (which also includes vertebrate viruses) and *Tospoviridae* as the most abundant plant virus families[17], both of which are potential threats to economically important crops.

We also identified seven RNA viral families associated with animals: *Nodaviridae* (71), *Astroviridae* (3), *Flaviviridae* (3), *Hepeviridae* (2), *Phenuiviridae* (4), *Reoviridae* (3), and *Rhabdoviridae* (2) (Fig. S9; Tables S4&S10). Additionally, two invertebrate RNA virus families were detected: *Dicistroviridae* (35) and *Iflaviridae* (8). Wen et al. further reported three vertebrate virus families—*Paramyxoviridae*, *Parvoviridae*, and *Togaviridae*[20]. However, unlike Wu et al.’s findings in Arctic permafrost soils[17], we did not detect RNA viruses linked to known human pathogens, such as *Nairoviridae*, *Hantavirus*, or *Coronaviridae*.

The RNA viruses identified in the TPC environment are potential pathogens for a range of hosts, including arthropods (*Dicistroviridae*), fish (*Nodaviridae*), birds (*Reoviridae*), and possibly humans (*Flaviviridae* and *Hepeviridae*). These viruses could cause diseases in their respective hosts, with potential ecological, economic, and public health implications. For example, *Hepeviridae* vContigs were assembled from wetland and cryoconite samples in Tanggula. The *Hepeviridae* detected in cryoconite may have been transported by wind and preserved on the glacier surface. Two vOTUs were identified within *Hepeviridae*. TGLSC3_k141_6817 represents a novel species, with its RdRp showing the highest similarity (52.25%) to *Hepelivirales* sp. (NCBI acc. WAY16491.1) from a water body in Berlin, Germany. It also shares similarities with Crustacea hepe-like virus (XGT97447.1, 33.13%) and bird RNA virus sequences (WKV33280, 40.35%). TGL_TJ1_k141_770407, another novel species, exhibits the highest RdRp similarity (40.19%) to *Hepelivirales* sp. (WAY16482.1), along with similarities to Clirnapec virus 81 (XII42435.1, 30.41%) and *Paslahepevirus balayani* (BBF24802.1, 30.59%).

The Tanggula glacier forefield wetland, located just 2–3 km from local herder settlements, serves as an important pasture. Although there is no direct evidence of their ability to infect humans, the relatively high abundance of some of these viruses (e.g., *Nodaviridae* and *Flaviviridae*) and their proximity to human settlements highlights the need for continued monitoring to evaluate potential risks.

### Metabolic potential of putative RNA virus hosts

RNA viruses infecting prokaryotes have been previously associated with phyla such as Bacteroidota[28, 33], Chloroflexota[28, 33], Pseudomonadota (formerly Proteobacteria) [33], *Candidatus Accumulibacter* sp. SK-02[34], and thermoacidophilic bacteria (e.g., *Hydrogenobaculum* sp. of *Aquificota*) [35]. Recent study by Wu et al. has also identified Actinomycetota and Cyanobacteriota as potential hosts for RNA viruses[28]. In this study, we predicted nine bacterial phyla as hosts for RNA viruses, including Bacillota (formerly Firmicutes; 13 MAGs), Bacteroidota (6), Pseudomonadota (6), Actinomycetota (3), Cyanobacteriota (2), Myxococcota (2), Fibrobacterota (1), Fusobacteriota (1), and Patescibacteria (1) (Fig. 1F; Fig. S10; Table S6). Additionally, we identified a link between an RNA virus and the archaeal phylum Halobacteriota (1) (Fig. 1F; Table S6). Six of these phyla had not been previously recognized as RNA virus hosts (Fig. S10). Even within known phyla (e.g., Pseudomonadota), we identified novel bacterial lineages as potential hosts, such as MAGs belonging to the orders of *SZUA-152* and *JABSOH01* (Fig. S10; Table S6).

The predicted prokaryotic RNA virus hosts exhibited diverse metabolic potentials, including sulfur metabolism, nitrogen metabolism, photosynthesis, carbon fixation, and carbohydrate degradation (Fig. S16). Notably, we identified two Cyanobacteriota MAGs linked to RNA viruses: one vContig from proglacial lake sediments (1QY1_k141_89640) and another from cryoconite (2006QYC3_k141_116683). We also detected potential cyanobacteria RNA viruses with AMGs in cryoconite (discussed below). Recent findings by Wu et al. suggest that RNA viruses in the high-altitude Lake Nam Co may also infect Cyanobacteriota[28]. These results imply that RNA viruses could influence photosynthesis, carbon fixation, and nitrogen cycling in TPC glacier and lake ecosystems. Taken together, our study significantly expands the known diversity and functional roles of prokaryotic RNA virus hosts, underscoring the previously overlooked ecological impacts of RNA viruses on TPC food webs and biogeochemical cycling.

### Expansion of prokaryotic RNA virus lineages

Previously, only three groups of RNA viruses infecting prokaryotes were known: the class *Leviviricetes* and the families *Cystoviridae* and *Picobirnaviridae* [36]. Recent studies, however, have expanded this list, identifying additional families with potential prokaryotic hosts in global oceans [37], global RNA virome dataset (RVMT)[38], and high-altitude lake water [28]. In this study, we identified vContigs from eight established ICTV families, seven RVMT family-level clades, and 18 unclassified clades (with ≥3 vContigs) that likely represent prokaryotic RNA viruses (Fig. S10; Tables S5&S6). These included families such as *Endornaviridae*, *Botourmiaviridae*, *Dicistroviridae*, *Tombusviridae*, *Virgaviridae*, *Marnaviridae*, *Narnaviridae*, and *Polymycoviridae*, previously thought to infect eukaryotes, as well as RVMT-established family-level clades like f.0156, f.0386, f.0026.base-Dicistro, f.0033, f.0206, and f.0296 etc. (Fig. S10C; Tables S5&S6). Among these, *Marnaviridae*, *Narnaviridae*, and f.0296 were also reported by Wu et al., further supporting their association with prokaryotic hosts [39]. The remaining 12 families and 10 unclassified clades were proposed as putative prokaryotes RNA viruses here for the first time.

The ongoing discovery of potential prokaryotic RNA viruses in previously unrecognized taxonomic groups supporting that the diversity of prokaryotic RNA viruses are largely underestimated[36]. The median RPM of each habitat of extended prokaryotic RNA vContigs is shown in Fig. S10C. Some vContigs of these families or clades showed moderate median RPM values across habitats, for example, f.0386 in snow (28.2), ice (15.9), wetland (14.6) and upland (12.5), suggesting a potential role in modulating microbial community dynamics.

### Linking RNA viruses to their potential hosts through AMGs

AMGs are acquired by RNA viruses through horizontal gene transfer from their hosts during infection, making AMGs valuable markers for identifying potential hosts of viruses[40-42]. For example, the *psbA* gene encoded by the snow RNA vContig (TGL216SS_k141_486099) of f.0102.base-Curvula (*Durnavirales*) exhibited >94% similarity to the D1 protein of algae (e.g., *Xanthophyceae*) and clustered closely with algal sequences in the phylogenetic tree (Fig. S13). This strongly suggests that the host of this snow RNA virus is likely a snow algae species. Similarly, the *cpcB* gene, encoding a phycocyanin protein in the cryoconite RNA vContig (2006QYC2_k141_333847), was identical to sequences from Cyanobacteria (e.g., WP_193972088.1, unclassified *Microcoleaceae* sp.) (Table S9). This vContig belongs to the RNA phage family *Fiersviridae*, indicating that its host is likely a photoautotrophic cyanobacterium.

Additionally, the *trxA* gene, encoding a thioredoxin protein in the ice RNA vContig (TGL5300top_k141_724901), showed 97.4% similarity to *Sphingomonas* sp. (e.g., WP_056058473.1) (Table S9). This vContig belongs to the RNA phage family *f.0368.base-Atkins* (*Leviviricetes*), suggesting that its host is likely a heterotrophic *Sphingomonas* species. Intriguingly, the *atpB* gene, encoding an ATPase F0a protein in the snow RNA vContig (QY215SS_k141_188611), exhibited 90.2% similarity to *Rhodococcus antarcticus* (e.g., WP_265383704.1) (Table S9). This vContig belongs to f.0386 (*Lenarviricota*), a group with strong RNA phage signals (Fig. S10), leading us to propose *Rhodococcus* sp. as its potential host.

Similarly, the *rplD* gene, encoding the large subunit ribosomal protein L4, plays a critical role in protein synthesis. The L4 protein encoded by the *rplD* gene in the cryoconite RNA vContig (GLYSC2_k141_244580) showed the highest similarity (86.2%) to Rhodothermota bacteria (e.g., *Rubrivirga marina*, WP_056058473.1). However, since this vContig belongs to an unclassified RNA virus group (C1921, Table S5), we can only infer that its putative host is likely a Rhodothermota-associated bacterium (Fig. 1G). Based on these linkages, we propose that glacier RNA viruses may influence glacier melting by modulating host metabolism through AMGs (see the Main Text).

### ORF and domain analysis of putative AMGs

In addition to using DRAM and functional annotations of ORFs (e.g., hhsuit; see Supplementary Materials and Methods), we assessed the completeness of functional domains in putative AMGs by aligning their amino acid sequences with their closest cellular homologs from the NCBI-NR database (Figs. S17 and S18). Detailed alignment procedures, exemplified by *PsbA*, are provided in the supplementary methods. Of the 39 AMGs analyzed, 11 (28.2%) were partial sequences compared to their cellular homolog ORFs. These included phycobilisome protein (2006QYC2_k141_333847_2), inner membrane component of T3SS cytoplasmic domain (AW1-1_k141_425352_4), glucose-6-phosphate dehydrogenase (AJ-7_k141_1444729_1), ribosomal protein L3 (GLYSC2_k141_244580_1), ribosomal protein L4 (GLYSC2_k141_244580_2), acyl-CoA dehydrogenase (KQGRC1_k141_320723_2), Rho termination factor (QY215SS_k141_188611_2), ricin-type beta-trefoil lectin (QY215SS_k141_188611_6), putative tricarboxylic transport membrane protein (TGL_TJ3_k141_162580_4), ATP-dependent Clp protease (TGL_TJ3_k141_464371_3), and IMP dehydrogenase/GMP reductase (TGL5300down_k141_394333_3). These AMGs contained either a single full domain or multiple domains of their respective functional ORFs (Fig. S18). Consequently, their roles in cellular metabolic pathways require further experimental validation.

### Limitations of the study

Our study primarily relied on metatranscriptomic data and bioinformatic analyses, which introduces several limitations:

i) Prokaryotic RNA virus prediction. Current understanding of prokaryotic RNA viruses remains limited[43]. While we identified several RNA virus groups in TPC environments that may infect prokaryotes, experimental validation is lacking. Pure or enrichment cultures of these RNA viruses with their potential hosts, or functional analysis of key genes encoded by these viruses[44, 45], would help confirm the predicted virus-host linkages.

ii) Risk evaluation and validation. We assessed the pathogenic risks of TPC RNA viruses through host assignments and genome feature-based predictions. However, methodological constraints make it challenging to definitively predict their risks[46]. Although tools like machine learning can enhance environmental virus detection, biological and clinical studies are essential to validate their infectivity in plants, animals and humans.

iii) AMG function verification. Alongside previous studies [19, 40, 41], we identified various RNA virus AMGs. However, unlike DNA virus AMGs [47, 48], the functional roles of RNA virus AMGs remain experimentally unverified. Consequently, their ecological roles remain speculative. Experimental validation is crucial to understanding how these AMGs influence host metabolism and broader ecological processes.

iv) Drivers of TPC RNA virus communities. To better understand RNA virus diversity, function, and distribution, assessing their absolute abundance is critical. Our study did not employ methods such as internal standards[43] to quantify absolute virus abundance, limiting our ability to quantify environmental RNA viruses. Additionally, while we described the biogeographic distribution of RNA viruses across TPC habitats, a systematic evaluation of environmental drivers (e.g., edaphic and climatic factors) was hindered by limited environmental data. Thus, our understanding of the factors shaping RNA virus communities remains incomplete. Future studies addressing these gaps will improve our knowledge of RNA virus diversity, function, and responses to climate change.

Supplementary Materials and Methods

**Samples collection and preparation**

The 79 samples were collected from habitats of glaciers (28 samples, covering habitats of snow, ice and cryoconites), proglacial lake (6), upland soil (16) and wetland soil (29) in permafrost regions from September 2019 to July 2023 (Fig. 1; Table S1). Snow (5), ice (5), and cryoconite (18) samples were collected from nine glaciers including Guliya glacier (GLY), Jiemayangzong glacier (JMYZ), Mugagangqiong glacier (MGGQ), Tanggula glacier (TGL), Qiangyong glacier (QY), Kuoqionggangri glacier (KQGR), Parlung No.4 glacier (PL), 24K glacier (24K), Zhuxigou glacier (ZXG). Proglacial lake sediments (6) were collected from Qiangyong lake (QY) which was mainly fed by the melting water from the Qiangyong glacier [27]. Upland soils were collected (16, with 13 from the surface and 3 from the subsurface) from Aerjin (AJ), the Altun Mountains National Nature Reserve and in permafrost regions of the Tuotuohe (TTH), the source region of the Yangtze River [49]. Wetland soils (29) were collected from the shore of one lake in AJ (5) and the permafrost regions of Longxiazai, Tanggula glacier (24).

Snow and ice samples (~5 cm deep) were collected within a square meter area using a pre-cleaned steel scoop and ice axe and then placed into 2 L sterile Whirl-Pak bags (Nasco, Fort Atkinson, USA) as described in the previous study [50]. For surface snow and ice, 2 mm of the sample was scraped with a sterile scalpel for decontamination. The cryoconite samples were collected from holes using a stainless-steel scoop and placed in pre-cleaned 1 L polycarbonate bottles (Nalgene, Thermo Fisher, USA). Proglacial lake sediments were collected with grabbers from the surface of the sediments (~15 cm) in QY lake and then transferred into 500 mL sterile Whirl-Pak bags (Nasco, Fort Atkinson, USA). Wetland soil samples were collected in the lakeside of AJ and permafrost regions of Longxiazai with pre-clean spades. The surface soil of each site was stored in three 500 mL sterile Whirl-Pak bags (Nasco, Fort Atkinson, USA). Upland soil samples were collected in permafrost regions of the TTH. In total, thirteen soil cores were sampled with a motor-driven soil column cylinder. Thirteen surface (0–20 cm) soil samples and three sub-surface (50–120 cm) soil layers of one soil column were collected and placed into 500 mL sterile Whirl-Pak bags (Nasco, Fort Atkinson, USA). All samples were stored at -20°C during the transportation from the field to the laboratory in Lhasa City (Tibet, China). Snow and ice samples were de-frozen at 4°C at dark and then filtered through 0.2 µm polycarbonate filters (Millipore) immediately. All samples were stored at –80°C or liquid nitrogen until total RNA extraction.

**Total RNA extraction, quality checking, sequencing, and read processing**

Total RNA was extracted within half a year after sampling. Total RNA was extracted from all samples with the RNAeasy PowerSoil Total RNA Kit (QIAGEN, Hilden, Germany) according to the manufacturer’s instructions. Residual DNA removal and RNA cleaning were carried out using DNase I and the RNA Clean&Concentrator-5 (Zymo, Irvine, USA), respectively. The quantity of total RNA was measured using RNA HS Assay Kit (Thermo Fisher Scientific, Waltham, USA) with Qubit 3 (Invitrogen, Carlsbad, USA). The quality of RNA was assessed using Bioanalyzer 2100 (Agilent, Santa Clara, USA). Ribosomal RNA (rRNA) was removed with Ribo-off rRNA Depletion Kit V2 kits (Vazyme, Nanjing, China). Libraries were prepared with VAHTS Universal V6 RNA-seq Library Prep Kit (Vazyme, Nanjing, China) for Illumina following the manufacturer’s recommendations. Sequencing was carried out using the Illumina NovaSeq 6000 platform with pair-end 2×150 mode by Guangdong Magigene Biotechnology Co. Ltd. (Guangzhou, China) company.

In total, over 4.16 TB of raw reads were obtained, with an average of 350 million raw reads per sample (Table S1). Raw reads were trimmed with Trimmomatic v0.39[51] with default parameters, which yielded 3.78 TB clean reads. The remaining rRNA reads were removed from total RNA with SortMeRNA v4.3.6[52], which yielded 2.6 TB non-rRNA reads, with an average of 223 million mRNA reads per sample.

**Metatranscriptomic reads assembly, RNA virus identification and verification**

mRNA reads were *de novo* assembled into contigs using MEGAHIT v1.29[53] with default parameters as previously described[37]. All RNA viruses of the kingdom *Orthornavirae* [hence, excluding satellite RNAs (realm *Ribozyviria*) and reverse-transcribing RNA viruses (kingdom *Pararnavirae*)] share a single hallmark protein, the RNA-dependent RNA polymerase (RdRp)[54]. Therefore, we searched for RNA viral genomes through mining assembled contigs encoding RdRps. Contigs with length ≥ 1 kb were kept for gene prediction with Prodigal v2.6.3[55] with the “-p meta” option setting and the default translation table. The yield protein sequences were subject to RdRp domain identification similar to previous studies by using hidden Markov model (HMMs) and hmmsearch (HMMER v3.3.2)[56] approaches. To increase the detection of divergent target domain sequences, HMMs from the *Tara* Oceans RNA virus dataset[19, 37], NeoRdRp pipeline[57], and the RVMT global RNA virome dataset[33] were used for RdRp domain search. The protein sequence containing domains with an E-value ≤0.01 (24,712) was kept for RdRp verification through the Palmscan algorithm[58] and metagenomic reads mapping strategy as described by Hou et al.[30] to remove DNA remnants (Table S11). RdRp sequences passed the two verification processes were confirmed as true positive RdRps and contigs containing these RdRps were considered as *bona fide* RNA viral genomes.

First, all protein sequences with putative RdRp domains were subjected to the Palmscan algorithm[58] screening based on PALMdb. Palmprint is a segment of the palm sub-domain robustly delineated by well-conserved catalytic motif. Sequences with a palmprint score ≥20 were treated as positive RdRp sequences. After Palmscan, 8,799 putative RNA viral genomes were kept, which is about 35.6% of the number from the above hmmsearch step. Second, we applied a metagenomic reads mapping strategy as described by Hou et al. [30] to remove DNA remnants since many of the metatranscriptomes were accompanied by metagenome from the same sample (Table S11; see below for metagenome sequencing). In addition, for metatranscriptome without accompanied metagenomes, sequences from the metagenome of similar samples (e.g., cryoconite) were used (Table S11). Metagenome reads were mapped against the RdRp nucleotide sequences from prodigal with Bowtie2 v2.4.255 with the “end-to-end” setting to check whether there was a DNA counterpart [30]. To avoid false negatives (true RdRps that were treated as DNA contaminants) caused by reads mapping on conserved regions, we set a moderate threshold for DNA reads mapping, which requires a horizontal coverage of 75% and a minimum vertical coverage depth of 1×. Zero sequence met the criteria of metagenome reads mapping. Therefore, 8,799 RNA viral genomes (vContigs) from all samples were obtained, with an average of ~111 vContigs per sample (Table S2).

**vContigs quality assessment**

The quality of the vContigs was assessed by using CheckV v1.01[59] with default parameters. To evaluate the completeness of RdRp domains, vContigs were translated to amino acid (AA) sequences by using transeq in EMBOSS v6.5.7.0 with six possible frames and all translational codes with transeq to resolve difficulties associated with alternative genetic code usage and non-canonical translation events. All translated AA sequences were searched against the 65 RdRp HMM profiles from Wolf et al.[60]. The aligning regions of each AA sequence with the highest score and the longest length were extracted as the best RdRp domain. The frame and code combinations from the AA sequence with the best RdRp domain were kept for genome annotation of the corresponding RNA virus (see below). The AA sequences with aligning regions of ≥90% of the average length of the best-matching HMMs were considered to contain “complete” RdRp domains [37].

**The generation of viral operational taxonomic units (vOTUs) of RNA viruses**

Generally, virus operational taxonomic units (vOTUs) represent the “species” rank of RNA viruses [19, 33, 37]. In this study, 8,799 RdRp domain sequences were clustered into 5,333 vOTUs using CD-HIT v4.8.1[61], with a clustering threshold of 90% amino acid identity (AAI) and default parameters as previously described[19, 30, 33, 37]. In addition, 8,799 vContigs nucleotide sequences were clustered into the 6,620 clusters (C90) by using CD-HIT v4.8.1, with a clustering threshold of 90% average nucleotide identity (ANI) and 80% alignment fraction (AF)[19, 33, 37].

**Taxonomy classification of TPC RNA viruses**

To assign taxonomic information for RNA viruses, we applied pipelines including RdRp AAI and vContigs ANI comparison as described by Neri et al.[33], Markov Cluster Algorithm (MCL) clustering and network analysis as described by Zayed et al.[37] and RdRp phylogenetic tree construction[33, 37]. Public reference databases, including the global RNA virome dataset (RVMT)[33], the *Tara* Oceans datasets (TO)[37], and IMG/VR-v4 database[62] were used along with the pipelines for taxonomy classification of TPC RNA viruses since these database or dataset have constructed dedicated and detailed RNA virus taxonomic classification framework.

First, we determine the taxonomy of each vContig based on the sequence identity against reference datasets (the RVMT, TO) or databases (IMG/VR-v4) with dedicated and detailed RNA virus taxonomic classification. According to the identity between query sequence with reference sequences, different taxonomy level of reference sequences was assigned to TPC vContigs as follows:

Level 1. Species level classification based on RdRp AAI and vContigs ANI as Neri et al.[33] consists of (1) vContigs encoding RdRps with exceptionally high amino acid identity to RdRps from the RVMT and TO datasets (via best BLASTp match with Identity 90%, Query-Coverage 75%, and E-value <1e-3); (2) vContigs with high nucleic similarity to vContigs from RVMT and TO dataset (95% ANI over 95% AF or Nident R 900 nt and E-value <1e-3, established using CheckV anicalc.py and aniclust.py scripts[59]); (3) vContigs sharing high nucleic similarity to those vContigs from RVMT, TO and IMG/VR-v4 database (via best dc-MEGABLAST hit at Identity 90%, Query-Coverage 75% OR Nident 900nt and E-value <1e-3). vContigs meeting these criteria were assigned species-level taxonomy based on their best database match.

Level 2. Family to genus level classification based on RdRp AAI consists of vContigs encoding RdRp domain sharing high amino acid similarity to those RdRps from the reference database (via USEARCH v10.0.240, UCLUST algorithm with Identity 50%, Query-Coverage 50%). vContigs meeting these criteria were assigned family-level taxonomy based on reference sequences.

Level 3. Phylum-level classification based on complete RdRps and MCL clustering. The completeness of RdRp domain sequences of the RVMT dataset[33] was first evaluated as described above, only “Complete” RdRp domain sequences (n=106,561) were kept. Then the “complete” (n=6,238) centroids RdRp domain sequences used by Zayed et al.[37] were downloaded for Markov Cluster Algorithm (MCL) clustering. The “complete” RdRp domain sequences (n=3,206) from the TPC dataset were combined with the two “complete” RdRp domain sequences dataset mentioned above and subject to clustering by using USEARCH v10.0.240[63] with the following parameters: usearch --cluster_fast -id 0.50 -sort length. The centroids of the resultant 21,763 clusters (638 from TPC datasets) were then used for MCL network analysis as previously described[37]. In brief, all centroid sequences were cross-compared running all-against-all pairwise blastp v2.13 with parameters (-gapopen 9 -gapextend 1 -word_size 3). E-values for each pair were extracted and negative-log10-transformed in MCL v14.137[64] (--stream-mirror --stream-neg-log10 -stream-tf ‘ceil(200)’). Inflation values of 1.1 were used to delineate Phylum level clusters as previously described [37]. With Inflation values of 1.1, a total of 237 phylum-level clusters were formed. All those TPC clusters contained RdRp domain sequences from RVMT, indicating full-length RdRps of TPC were covered by the RVMT dataset at the phylum level.

Taxonomic confidence followed the hierarchy: Level 1 > Level 2 > Level 3. Higher-confidence classifications were prioritized. Number of vContigs belonging to Level 1, 2, and 3 were 3,013, 3,418, and 462, respectively. For all RNA viruses within each established phylum, a phylogenetic tree of RdRps was constructed to verify the taxonomic classification (see below). Finally, for RNA viruses without a taxonomic classification from Level 1 to Level 3 (1,906 vContigs), we also carried out palmprint similarity analysis on RdRps with the Serratus palmprint database (Palmdb)[58] to get putative phylum-class level classification and BLASTp (score >45, e <0.00005) to all reference databases (including TO, RVMT, Serratus and LucaProt) to get the most closely associated RdRp sequences in the reference database (Table S12).

**Phylogenetic analysis of the RdRp amino acid sequences**

We constructed phylogenetic trees for all RdRp amino acid (AA) sequences classified into 6 established RNA viral phyla to verify the classification. Reference sequences were obtained from RVMT C90 and TO during the classification step (see above). In brief, the RdRp AA sequences from each dataset or database that clustered with our RdRps at each level were kept as references. Reference RdRps and TPC RdRps from the same phylum were pooled together and aligned using MAFFT v7.508[65] with default parameters. The multiple sequence alignment was filtered using trimAl v1.4.rev15[66] with -gappyout parameters and custom scripts (fasta_drop.py) that remove sequences with >70% gaps. The final multiple sequence alignments were used for tree construction. The maximum-likelihood tree was constructed by using the FastTree software v2.1.11 with the parameters “-gamma -lg -boot 1000”. The tree was visualized with iTOL[67].

**Comparative analyses of TPC RNA viruses**

We compared the RNA vContigs and RdRp domains of this study with the ones from four reference databases, including the TO[19], the RVMT[33], the LucaProt[30], and the Serratus[68]. The novelty was estimated through two approaches:

i) RdRp based. To compare our data with published dataset at phylum level, “complete” RdRps from TPC, RVMT and TO were clustered by using USEARCH v10.0.240 (-id 0.5, -qcov 0.5) and MCL clustering (inflation values of 1.1).

Meanwhile, we clustered the RdRps of vOTUs with the RdRps from RVMT, LucaProt, TO and Serratus datasets by using CD-HIT v4.8.1[37, 61]. vOTUs with a RdRp AAI less than 90% to RdRps from the four datasets were defined as “unique” vOTUs as shown in Fig. 1C and Table S2.

ii) RNA virus genome-based. As a complement, we also carried out genome sequence comparisons as described by [19, 37]. Nucleotide sequences of vContigs from the TPC also were clustered with those from RVMT, LucaProt and TO by using USEARCH v10.0.240 and UCLUST algorithm with parameters: -id 0.9 and -qcov 0.5.

**Genomic features and function annotation of TPC RNA viruses**

Functional annotation of TPC RNA viruses was performed using three complementary approaches including i) ORFs based on default code; ii) ORFs based on optimized code; and iii) Based on amino acid sequences of functional domains (Fig. S19).

i) ORFs based on default code. A total of 8,799 nucleotide sequences of vContigs were annotated by InterProScan v5.62-94.0[69] against MobiDBLite (v2.0)[70], PRINTS (v.42.0)[71], Phobius (v.1.0.1)[72] and TMHMM (v.2.0c) database[73].

ii) ORFs based on optimized code. Optimize code was determined by the longest RdRp and corresponding code table as described in the vContigs quality assessment section. For RNA viral genome domain annotation, ORFs were identified by using Prodigal v2.6.3 with the optimized genetic code. One iteration of hhblits (-n 1 -e 0.001) by HHsuite v3.3.0[74] and UniRef30_2020_03 database[75] was used to generate profiles for amino-acid sequences. Generated profiles were searched against Pfam 35 to identify domains in the amino-acid sequences, and hits with >95% probability score were used for domain annotation[37].

iii) Based on amino acid sequences of functional domains. Since the determination of code usage is still a challenge, we also applied a functional domain annotation on the translated virus genome sequences with the longest RdRp domain detected without ORF calling. AA sequences from contigs translated by EMBOSS transeq of each vContigs were annotated by hmmsearch (from the HMMER V3.3.2 suite)[76] to match these proteins to hidden Markov models (HMMs) gathered from multiple protein profile databases using a maximal E-value of 0.001 (PFam 35[77], CDD v.3.19 [78], Gene3D v4.3[79], ECOD 2017 release[80], LysDB[33]). LysDB is from a previous study[33], which contains a custom collection of HMMs profiles for proteins with bacteriolytic functions.

**Identification of Shine-Dalgarno (SD) sequences of RNA viruses**

Two pipelines were used to identify the ribosome binding site (RBS) motif (Shine-Dalgarno sequences, SD) within each vContigs (Fig. S20), including OSTIR[81] and Prodigal[55]. The Shine-Dalgarno (SD) sequence is an RBS in bacterial and archaeal mRNA. The prevalence of SD in an RNA virus indicates it is more likely a prokaryotic RNA virus[33, 82]. OSTIR v1.1.0 was used to identify SD sequences of the virus with anti-Shine-Dalgarno sequences (aSD: 5’-ACCTCCTTT/A-3’). For Prodigal, default parameters were used. In addition, vContigs from the RVMT and TO were also included for SD sequence identification and comparison.

**Calculation of the vOTU relative abundance**

mRNA reads were mapped to the nucleotide sequences of the vOTU genomes with Bowtie2 v2.2.5 using the sensitive mode. The mapped reads were further filtered using Samtools v1.15.1 with -q 30 option to keep only high-quality mappings. Mapped reads per million total clean reads (RPM) values were used to represent the abundance of vOTUs. The RPM value of each vOTU within each sample was calculated according to the formula: RPM = Mapped Reads × 10^6^/Total Reads[30, 83, 84]. The coverage percentage of each vOTU was estimated by using CoverM v0.6.1 with -m covered_fraction option.

**The transcript abundances of MAGs and host functional genes**

The transcript abundances of MAGs were estimated by using BBmap v39.01 and coverM with ‘genome’ module with default parameters. Host marker genes were identified by using hmmsearch. Clean mRNA reads were mapped to host the nucleotide sequences and transcripts per million mapped reads (TPM) was generated based on the sorted bam files using coverM with ‘--min-covered-fraction 10 -m tpm’ parameters, as previously described [85, 86].

We collected 15,640 medium-high quality MAGs (with completeness > 50% and contamination < 10%) from the glacier, lake sediment, wetland, and upland of the TPC[50]. All these MAGs were clustered into strain-level units via dRep v3.4.5 with ANI 99% threshold. A total of 6,063 strain-level MAGs were obtained and used to estimate transcript abundances. BBmap v39.01 and coverM with ‘genome’ module with default parameters were used to calculate the transcript abundance of MAGs. Briefly, clean mRNA reads were mapped to host MAG sequences with default parameters, and yielded bam files were sorted using Samtools v1.15.1. TPM was generated as described above.

To find the host marker genes (including both prokaryotic and eukaryotic *cox1* and *rps3*), we used an HMM profile from Pfam Cox1 (PF00115), and HMMs for (https://github.com/AJProbst/rpS3_trckr) Rps3 and searched using hmmsearch (-E 0.00001) from the HMMER suite as previously described[87]. The Cox1 protein sequences were dereplicated and clustered at 98% AAI representing an estimated species-level designation. The Rps3 protein sequences were clustered at 99% AAI, representing species-level differences using CD-HIT. The identified proteins were classified using the NCBI protein blast. Clean mRNA reads were mapped to host the nucleotide sequences representing *rps3* and *cox1* with default parameters, and yielded bam files were sorted using Samtools v1.15.1. TPM was generated as described above (Fig. S21).

**Inference of virus-host relationship**

To infer putative hosts of RNA viruses, we employed six approaches:

i) Taxonomy based. The family of RNA viruses was used to retrieve information on putative hosts according to ICTV metadata (<https://ictv.global/vmr>; MSL39.v4) and RVMT meta-information (https://riboviria.org/).

ii) CRISPR-spacer screening. We retrieved 52,134 CRISPR spacer sequences from 15,640 medium-high quality MAGs of the TPC. Identification of the CRISPR spacer was performed by MinCED v0.4.2 with default parameters. In addition, a CRISPR-Cas spacer database of iPHoP_db_Sept21_rw compiled from GTDB, IMG, and GEM v1 database with 1,398,130 CRISPR-Cas spacers was also included for virus-host prediction [88]. All spacers were queried for matches against vContigs using the BLASTn-short function implemented in the NCBI BLAST v2.13 package with parameters “-evalue 1e-10, -perc_identity 95, -dust no -word_size 7”, allowing only <= 1 mismatches and minimum identity of 90% (Table S6) across the entire length of the spacer[33, 89].

iii) Endogenous Viral Elements (EVEs) based. EVEs are nucleotide sequences derived from DNA or RNA viruses that have integrated into host genomes. For this third approach, all nucleotide sequences from cellular organisms available in NCBI GenBank release 243 were used as a nucleotide database. To avoid including exogenous RNA virus genomes in the database, we excluded sequences shorter than 45 kb since the longest RNA virus genome reported so far is 45 kb[30]. To assess the evolutionary relationship between vConitgs and EVEs, the near-complete RdRp protein sequences were searched against the nucleotide database by using tblastn algorithm. As previously described[90], the thresholds were set to 100 amino acids for alignment length and 1 × 10^−20^ for e-value.

iv) Co-occurrence network based on abundance[87]. Co-occurrence network analysis was used to reveal putative virus-host linkages. First, MAGs, *rps3* and *cox1* genes were filtered with a max TPM>1000 and detected in at least 10 samples with TPM>5 threshold and vContigs were filtered with detected in at least 10 samples with RPM>0 threshold. Then a co-occurrence network was built based on the abundance of filtered sequences with Spearman correlation algorithms. Only correlations with thresholds: r>0.7 and p <0.001 were kept as previously described[87] (Table S5). Relative abundance datasets were deposited in figshare (<https://figshare.com/s/949e8151580d648951a5>).

v) RNA virus hallmark genes. Based on functional annotation of RNA virus genomes (see above), RNA virus hallmark genes were retrieved, including the movement protein of plant for plant infecting RNA viruses, and phage maturation protein, lytic protein, pro_E2 and pro_ring proteins for prokaryotic RNA viruses (Table S5; see below for more details).

vi) iPHoP. The iPhoP v1.3.3 with default parameters [88] was used to predict prokaryotic viruses. Hosts with a score >90 were kept.

Finally, RNA viruses from previously known prokaryotic RNA virus taxonomic groups (e.g., *Steitzviridae*) according to ICTV and RVMT or with CRISPR-spacer linkage to MAGs were considered (Table S6) as confidential prokaryotic RNA viruses. To keep the stringency, remaining vContigs were only considered as credible prokaryotic RNA viruses if they have at least three of the following signals: 1) phage maturation protein; 2) MS2 Phage Coat Protein (MS2coat); 3) lytic protein; 4) pro_E2 and pro_ring proteins; 5) with 50% ORF containing RBS motifs (RBS supported); 6) iPHoP host prediction hits; 7) virus-host abundance correlation hits (Table S5). In total, we identified 180 vContigs belonging to previously unknown prokaryotic RNA viruses (Table S5).

**Taxonomy and function annotation of MAGs with RNA virus associations**

The 36 MAGs associated with RNA viruses were downloaded from NCBI RefSeq database or IMG database based on their accession number, which were then analyzed by using GTDB-Tk v2.4.0 (with database r220) [91] and CheckM2 [92] to update the taxonomy information and genome quality (Table S6). A phylogenomic tree was constructed as previously described by using FastTree v2.1.11 and multiple sequences alignment of marker genes from CheckM2 [93]. Then all MAGs were annotated using the Distilled and Refined Annotation of Metabolism (DRAM)[94] pipeline with default parameter as previously described[93]. DRAM is a tool for annotating genomes, and KOfam, UniRef-90, PFAM-A, dbCAN, RefSeq viral, VOGDB, and the MEROPS peptidase databases were used for homolog search and genome annotations in the current study (Fig. S16).

**Risk assessment of RNA viruses with Zoonotic**

In addition, zoonotic_rank v1.2.1[95] with default parameters was applied to identify and prioritize potential human-infecting viruses in the TPC RNA virus communities. Metadata files were generated for all CDS regions from 8,799 vContigs using Prodigal, followed by the execution of the PredictNovel.R script. A total of 8,653 vContigs met the quality threshold and proceeded to prediction (Table S7), and a total of 1,043 vContigs met the default calibrated_score threshold of 0.293, which were classified as potential zoonotic viruses with priority_category ranks of “high” (1,007, 11.4% of 8,799) and “very high” (36, 0.4%). To control the false positive rate, we retained only those vContigs with a priority_category rank of “very high” as putative zoonotic viruses (Table S7).

**Identification of AMGs in vContigs**

Auxiliary metabolic genes (AMGs) encoded by vContigs were mainly identified using functional annotation and manual checking as previously described[19]. All annotations from RNA viruses contigs annotation were screened for putative cellular metabolic functions.

To verify the AMGs encoded by RNA viruses were not from transcripts of cellular genes, we applied a similar strategy as RdRps through metagenome reads mapping against the nucleotide sequences of putative AMGs. Metagenomic clean reads were mapped against the nucleotide sequences of AMGs with Bowtie2 v2.2.5 with the “end-to-end” setting. The mapped reads were further filtered using Samtools v1.15.1 with parameter: -q 30 -F 0×08 -b -f 0×2. Originally, we obtained 48 putative AMGs. After metagenome reads mapping, 9 AMGs with horizontal coverage greater than 75% were considered as “contamination” from host transcripts and were removed from downstream analysis. Thirty-nine AMGs from 15 RNA vContigs were kept (Fig. S11; Table S8). Among them, thirty-five were first reported in this study and only four were previously reported [19, 96] (Table S8). In addition, the functions of these AMGs were further checked with Phyre^2^ as previously described[97]. For genomic architecture, the annotation files of vContigs with putative AMGs were visualized in Geneious v2021.2.2 (Fig. S11). In addition, a phylogenetic analysis of PsbA amino acid sequences was carried out to verify its origins (Fig. S13).

**Phylogenetic analysis of PsbA amino acid sequences**

Reference sequences were obtained from NCBI IPG database by searching with “photosystem II protein D1” and filtered out none PsbA domain using hmmsearch with TIGR01151.1.hmm and PF00124.hmm. The gathering score of 27 and length of 180 AA residue were used for filtering. After filtering, all reference PsbA AA sequences were dereplicated using CD-HIT with 0.99 as the cutoff. PsbA of snow RNA viruses and non-redundant reference PsbA were combined for muscle v5 alignment, trimal v1.4.rev15 (with -gappyout) and gaps removal (drop sequences with >70% gaps). The final multiple sequence alignments were used for maximum-likelihood tree construction using the FastTree v2.1.11 with the parameters “-gamma -lg -boot 1000”.

**Estimation of shared RNA viruses across different habitats**

To establish the linkage of RNA virus between different habitats, we applied two strategies:

i) The mRNA reads recruitment strategy. We define that a virus presents in one sample if it has a vContigs RPM value ≥10 within that sample[30]. Then it is straightforward that two habitats share the same RNA virus if vContigs of the species were present in the two samples of different habitats.

ii) Genome-based vOTUs sharing strategies. 90% of RdRp AAI (via CD-HIT clustering) represent shared vOTUs between different samples at “species”-levels, respectively. If one vOTU was shared between samples from different habitats, then this vOTU was defined as shared vOTUs between the two habitats.

**Enrichment analysis of RNA virus family and vOTUs**

Enriched RNA virus families were identified by their maximum RPM among habitats. If the maximum RPM of a family in a habitat was ≥ 4 times that of the second highest maximum RPM, we considered that family to be enriched.

**Statistics analysis and figure generation**

Alpha-diversity (Shannon and Richness) and beta-diversity analyses were carried out using vegan package in R v4.0.3 with the RPM values of vOTUs. The dissimilarity of RNA viral communities (beta-diversity) was estimated by vegan package in R v4.0.3 with Bray-Curtis distance calculated from the RPM matrix. The clustering of viral communities was visualized with non-metric multi-dimensional scaling (NMDS) with the RPM-transformed Bray-Curtis distance.

Statistics analyses like PERMANOVA were conducted in R using the ‘vegan’ package (v.2.5-7). Significant difference evaluation (e.g., DunnTest *post-hoc* test) was also performed using ‘FSA’ package (v0.9.5; https://fishr-core-team.github.io/FSA/). Manuscript figures were mainly generated in R sing ggplot2 and pheatmap (<https://github.com/raivokolde/pheatmap>) packages and custom R scripts. The figure format was adjusted using Adobe Illustrator if needed.

**DATA AVAILABILITY**

Metatranscriptomic datasets from the current study were deposited at NCBI with accession number of PRJNA1115801. The codes and programs used for analyses are available at <https://github.com/Hame1N/TPC-RNA-virus>. TPC RNA virus genome datasets, including the RdRps amino acid sequences, vContigs sequences, full functional annotations of the 8,799 RNA virus genomes were available at figshare (https://doi.org/10.6084/m9.figshare.c.7390684). In addition, Supplementary Tables S1-S12, Supplementary Figures S1-S21, alignment files for AMGs domain annotation related to Fig. S17 and S18 were also deposited at figshare with the same link.


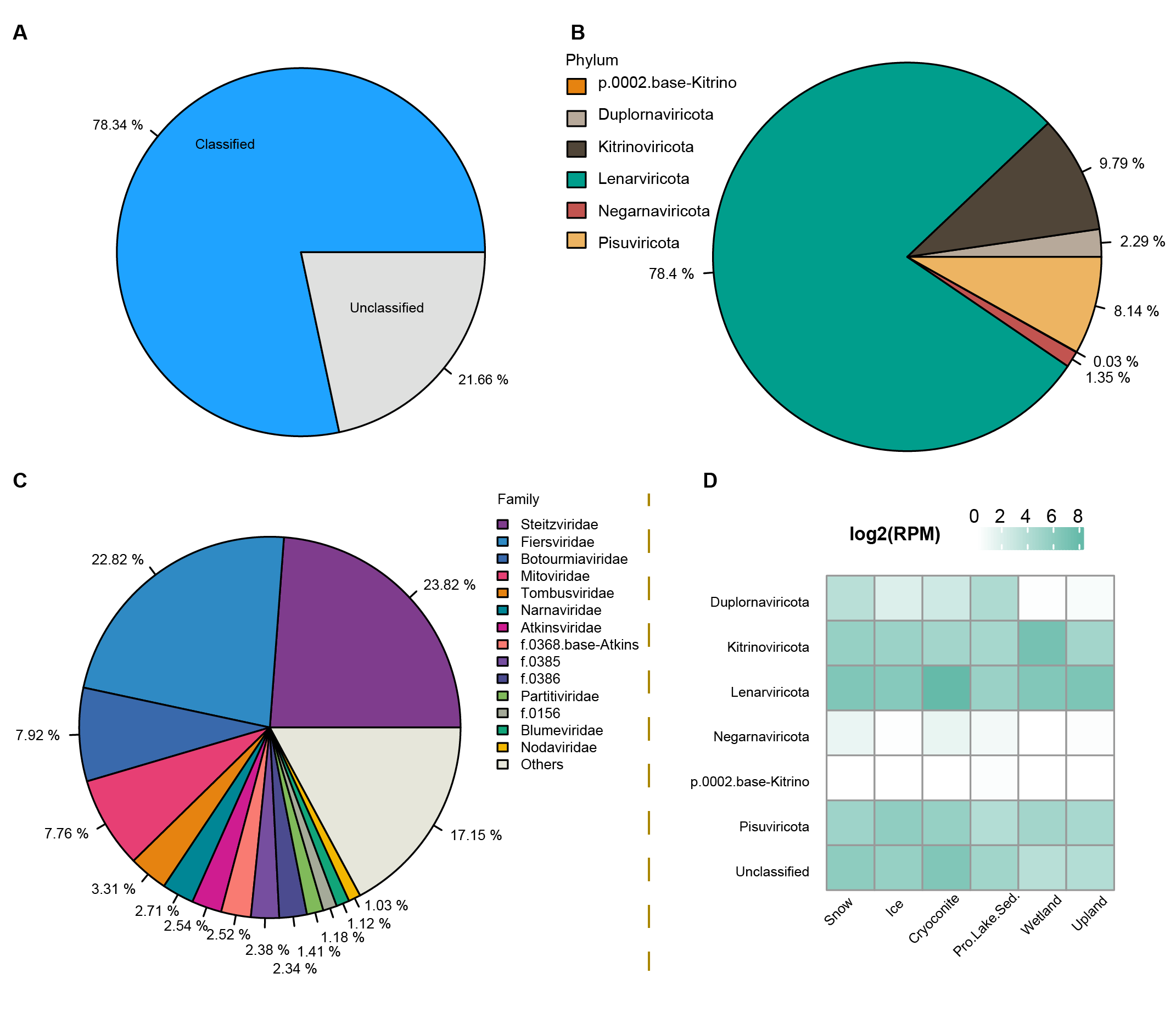


Fig. S1. Proportion of classified and unclassified RNA viral genomes and the relative abundance of vOTUs at Phylum-level.

**(A)** The proportion of classified and unclassified vContigs. **(B)** The proportion of vContigs belonging to established phylum, with the proportion of all classified vContigs scaled up to 100%. **(C)** The proportion of vContigs belonging to established dominant families, with the proportion of all classified vContigs scaled up to 100%. **(D)** The median RPM of each phylum across habitats.


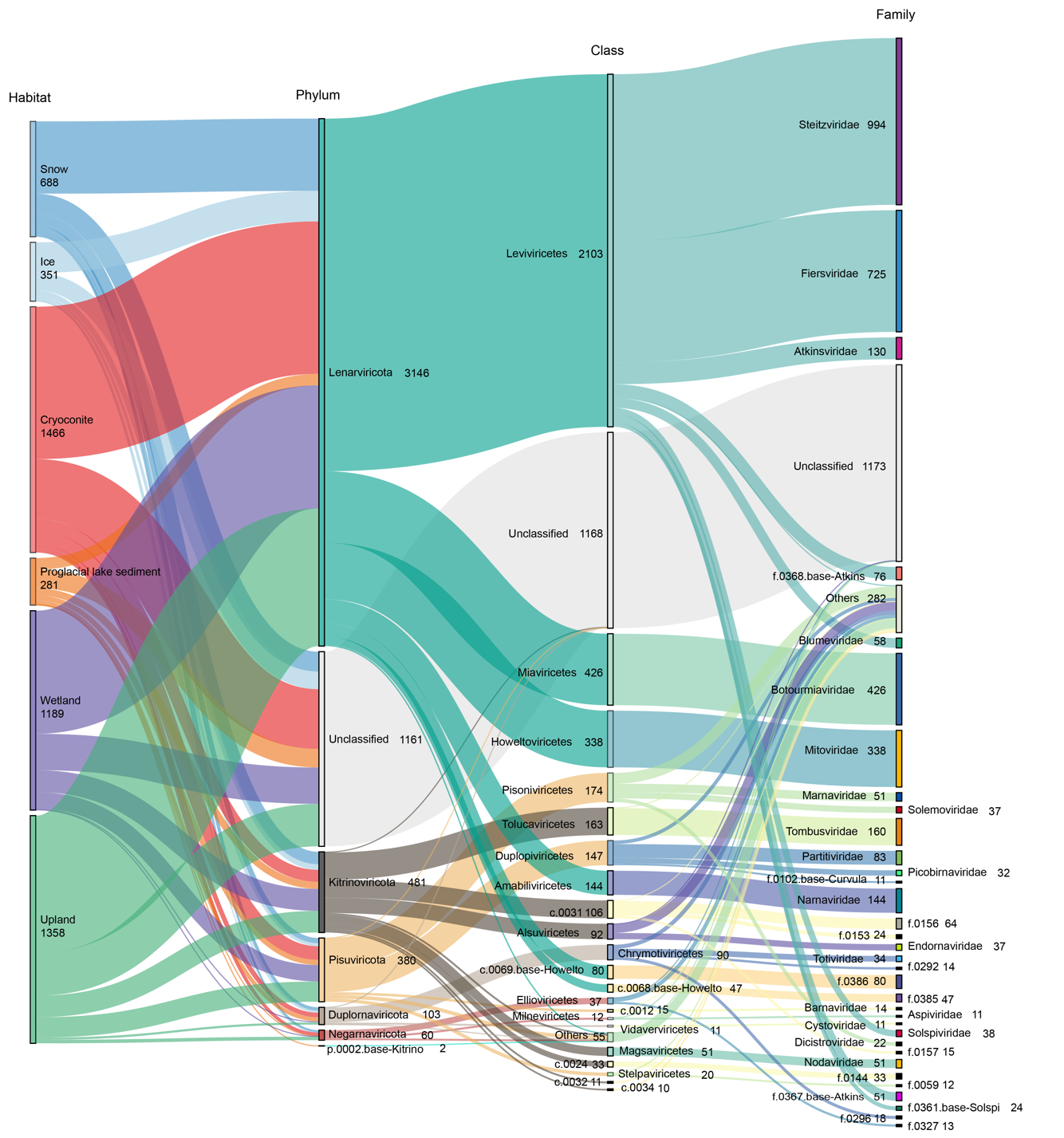


Fig. S2. Taxonomic affiliation of TPC RNA viruses.

Sanky plot showing the distribution of vOTUs in different RNA viral taxonomic ranks.

Families with vOTUs number ≥10 are shown and with the remaining grouped as Others. Numbers of vOTU belonging to each category are also shown after the category name. Unclassified means the taxonomy of RNA viruses could not be classified (See Table S3 and Supplementary materials and methods for more details).


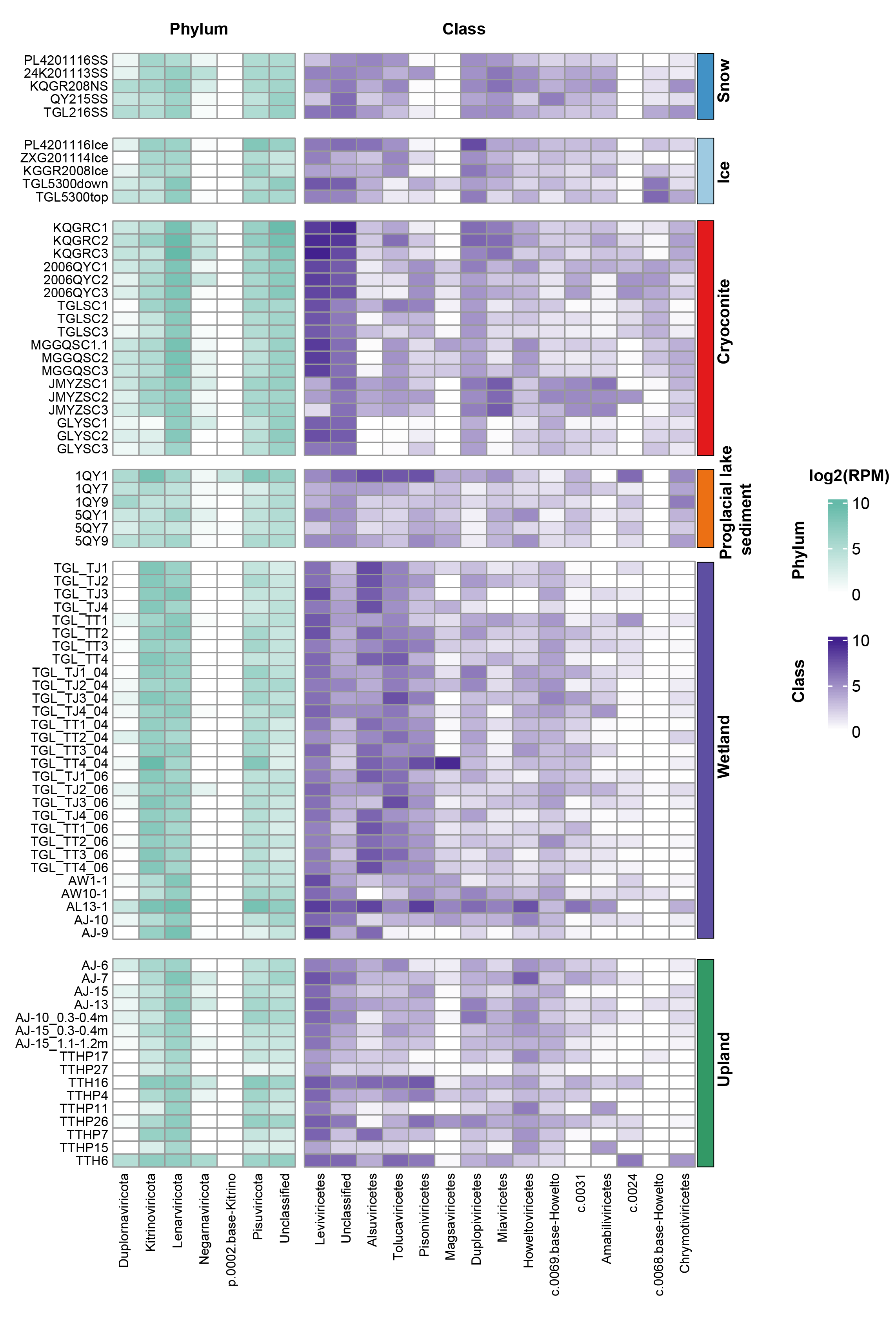


Fig. S3. The abundance of RNA viral Phylum and Class across samples.

The RPM of dominant RNA viral Phylum **(A)** and Class **(B)** across samples, respectively. Samples are grouped according to habitats.


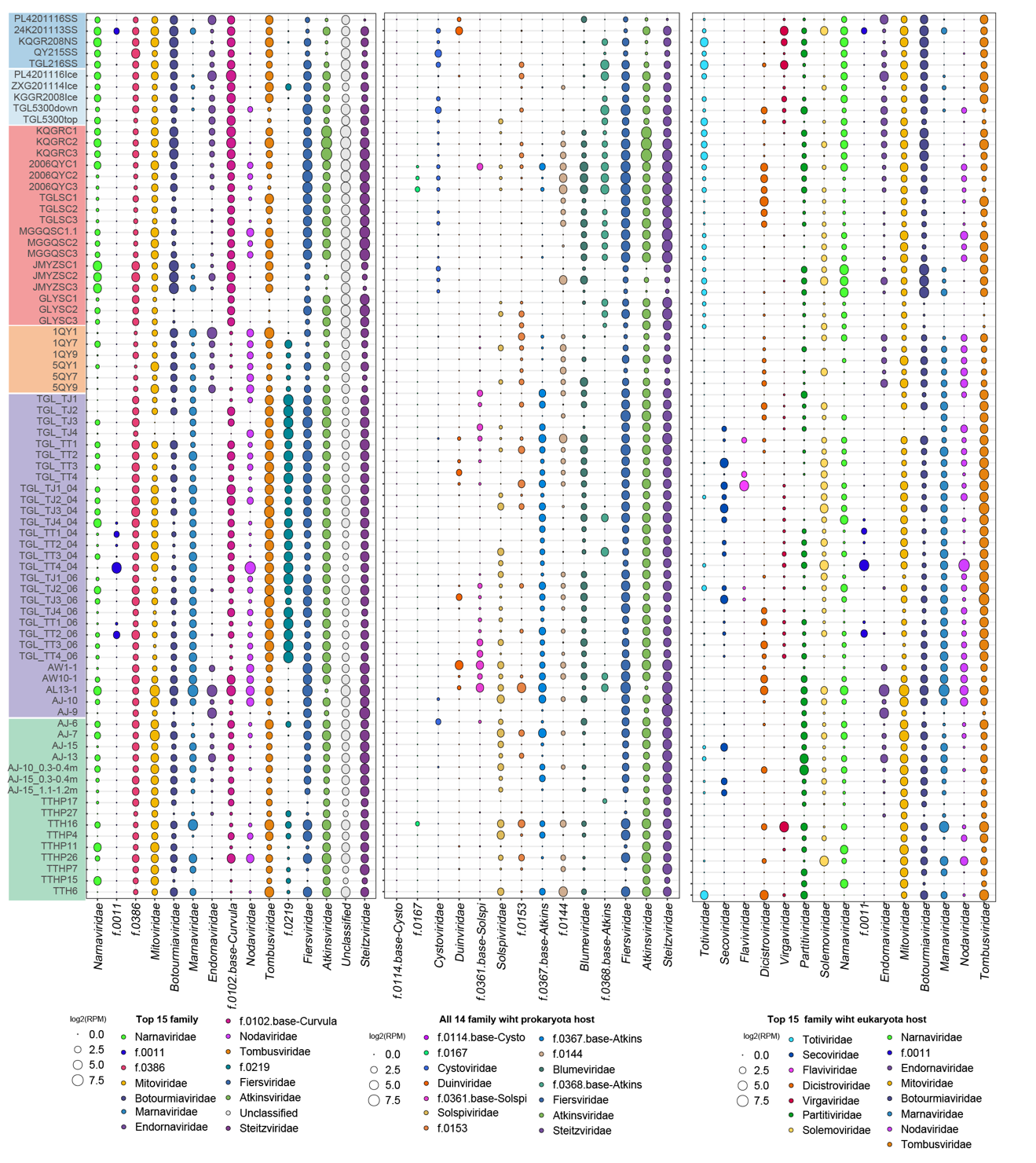


Fig. S4. RNA virus community structure of the TPC.

RPM of RNA virus families across different habitats. **(A)** RPM of the most abundance families (as shown in Fig. 1D) across different habitats. **(B)** RPM of prokaryotic RNA virus families. **(C)** RPM of eukaryotic RNA virus families. Samples are grouped according to habitats.

**
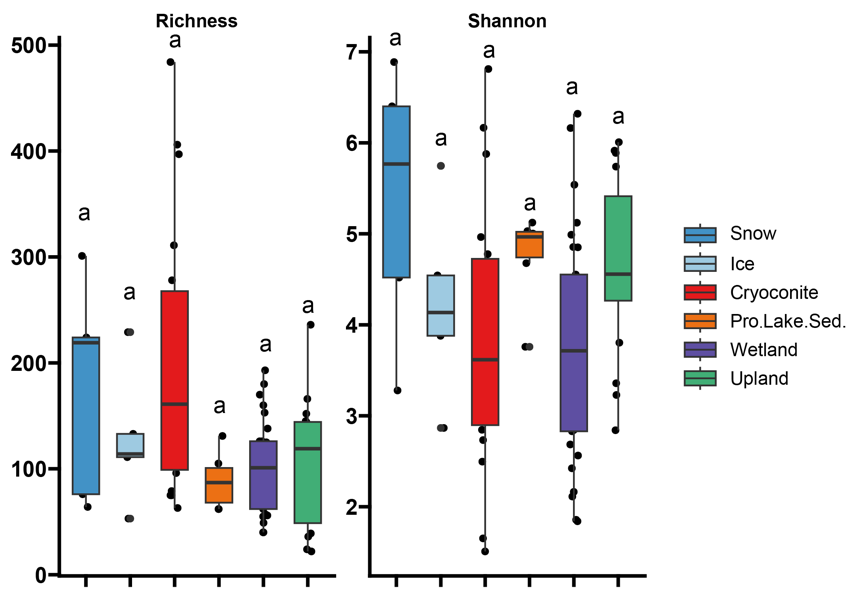
**

Fig. S5. Alpha-diversity of the TPC RNA virus communities.

Different letters above each box indicate significant differences (*P*<0.05, DunnTest) between habitats.

Fig. S6. R^2^ values of pair-wise adonis analysis of TPC RNA virus communities.

Red, *P*<0.001, black, *P*<0.05, dark blue, 0.05<*P*<0.1. Total R^2^ equals 0.237 for habitats, with *P*<0.001. See Fig. 1E in the main text.


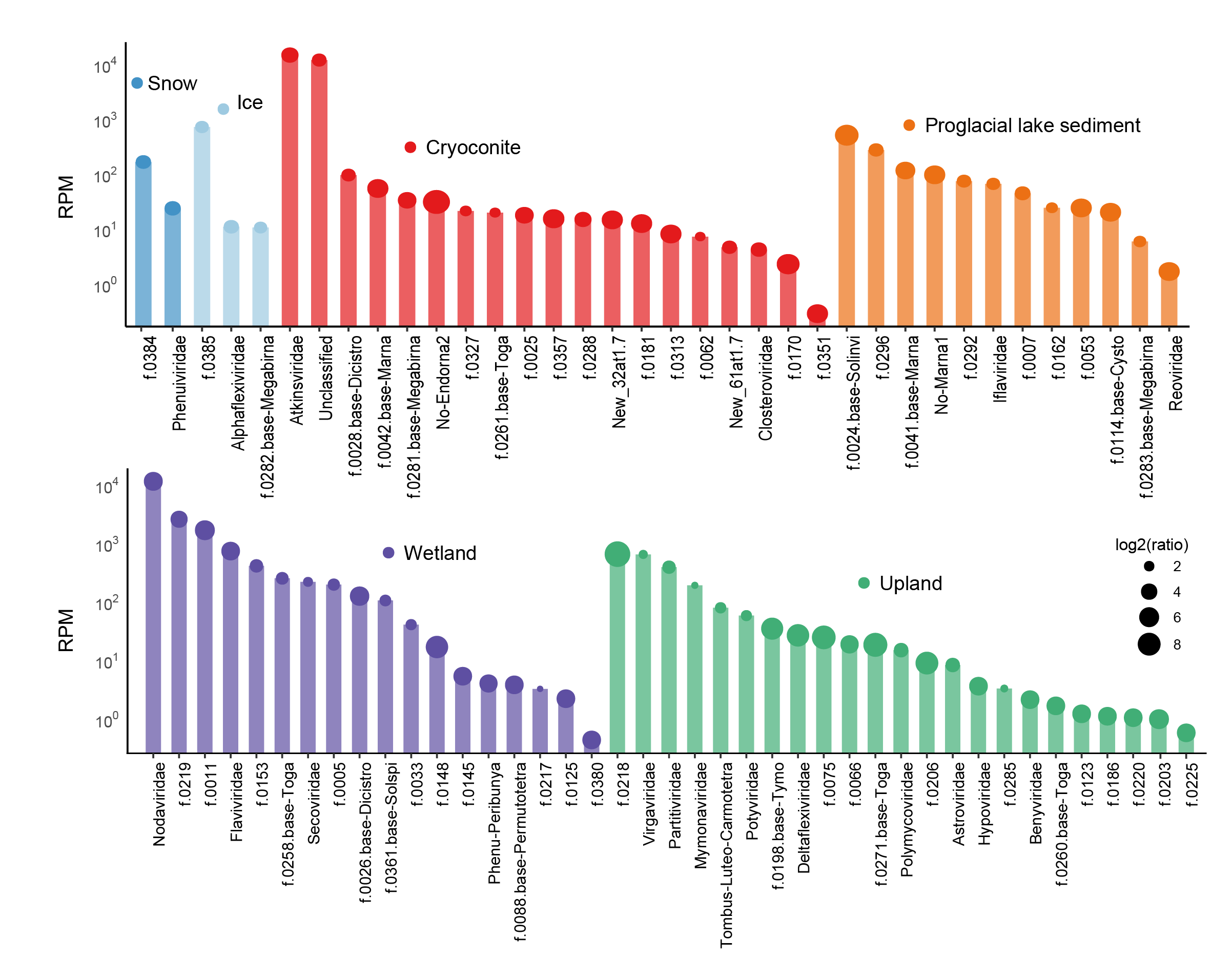


Fig. S7. Habitats enriched TPC RNA virus families.

Lollipop plot showing the maximum RPM of habitats enriched virus families and the environments in which they were enriched. The size of the dots on top of the bar indicates the folds of the RPM of the corresponding family in the enriched environments compared to that of the environment with the second highest values.


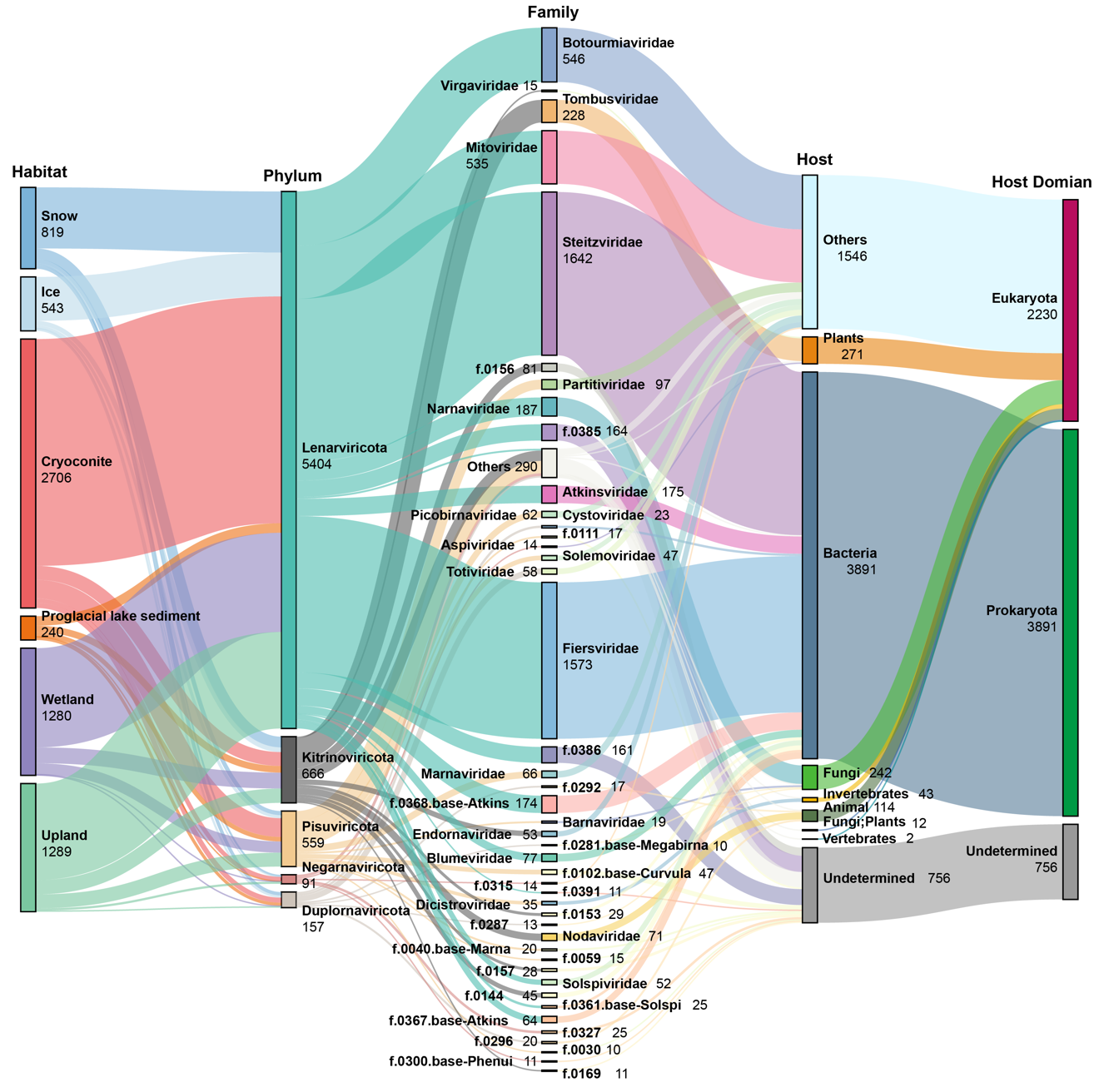


Fig. S8. Sankey plot showing the distribution of vContigs in habitats, phyla, families, and the types of their corresponding hosts.

Families with vContigs number ≥ 10 are shown and with the remaining grouped as Others. Undetermined means the host could not be assigned according to the taxonomy of RNA viruses. The consensus host information is shown here. For a full description of host information, see Table S4.

**
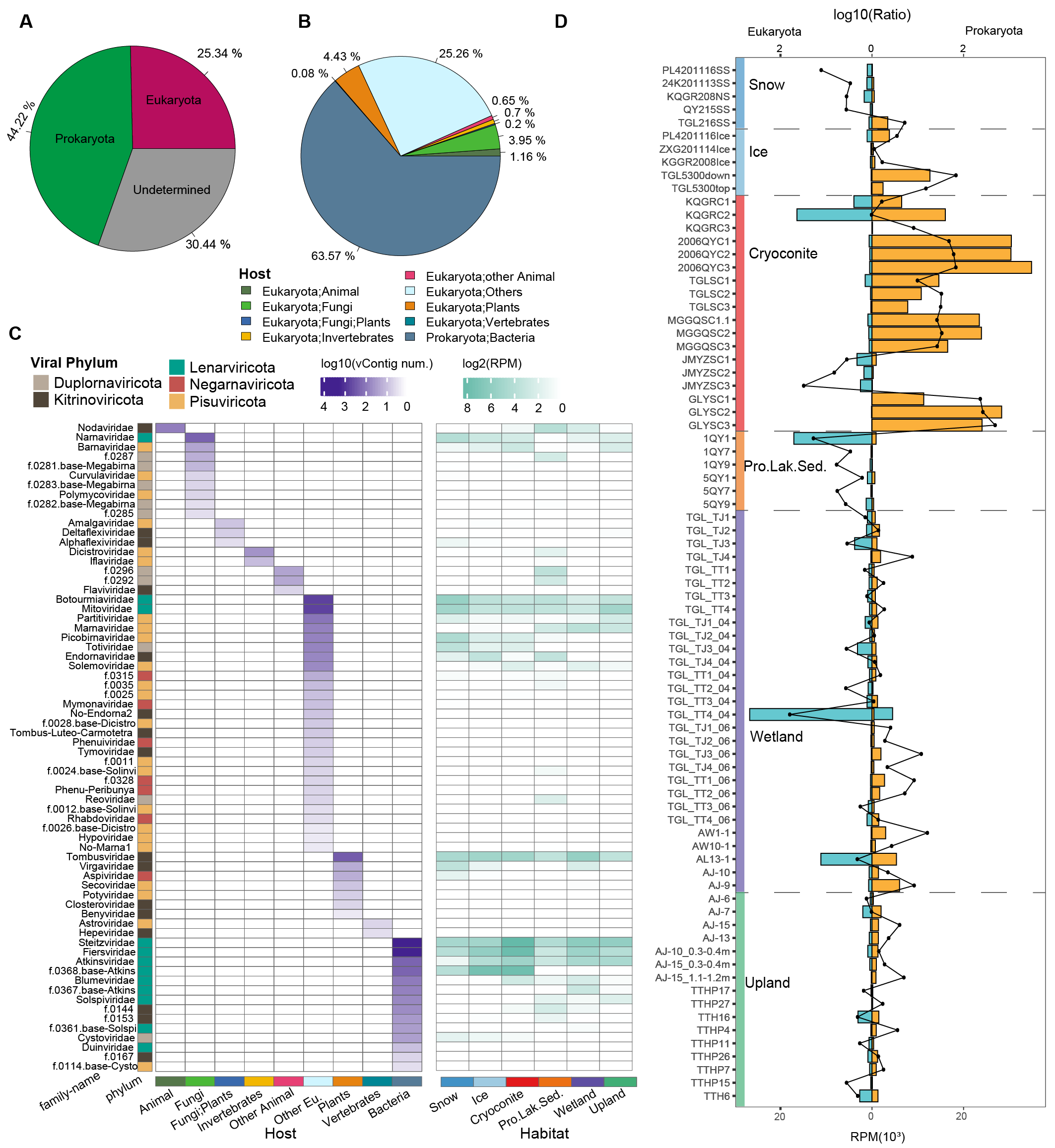
**

Fig. S9. Viral-host linkage of the TPC RNA viral communities.

**(A)** and **(B)** Composition of host assigned to the TPC vContigs based on vContigs taxonomic classification. Data in panel (B) correspond to the Eukaryota plus Prokayota fractions of panel (A), which were scaled up to 100% in (B). **(C)** Numbers of vContigs (left heatmap) and the relative abundance (right heatmap) of previously known eukaryotic and prokaryotic RNA viral families according to ICTV and RVMT. For families with multiple host assignments, a consensus description for potential hosts is shown. For example, *Astroviridae* could infect both vertebrates and arthropods (invertebrates), all belong to animals, hence “Animal” is used. *Reoviridae* which might infect both plants and vertebrates, “Eukaryota; Others” is assigned for it in the figure. “Fungi:Plants” denotes viral families with both Fungi and Plants infecting RNA viral families (e.g., *Alphaflexiviridae* and *Deltaflexiviridae*). Full list of potential hosts is provided in Table S4. Panels B and C shared the same color code for hosts. **(D)** Comparison of the RPM of eukaryotic and prokaryotic RNA viral families across samples. Bars indicate the RPM of eukaryotic (left) and prokaryotic (right) RNA viruses and dots indicate the ratio of the RPM between eukaryotic and prokaryotic RNA viruses. Panels C and D share the same color code for habitats.


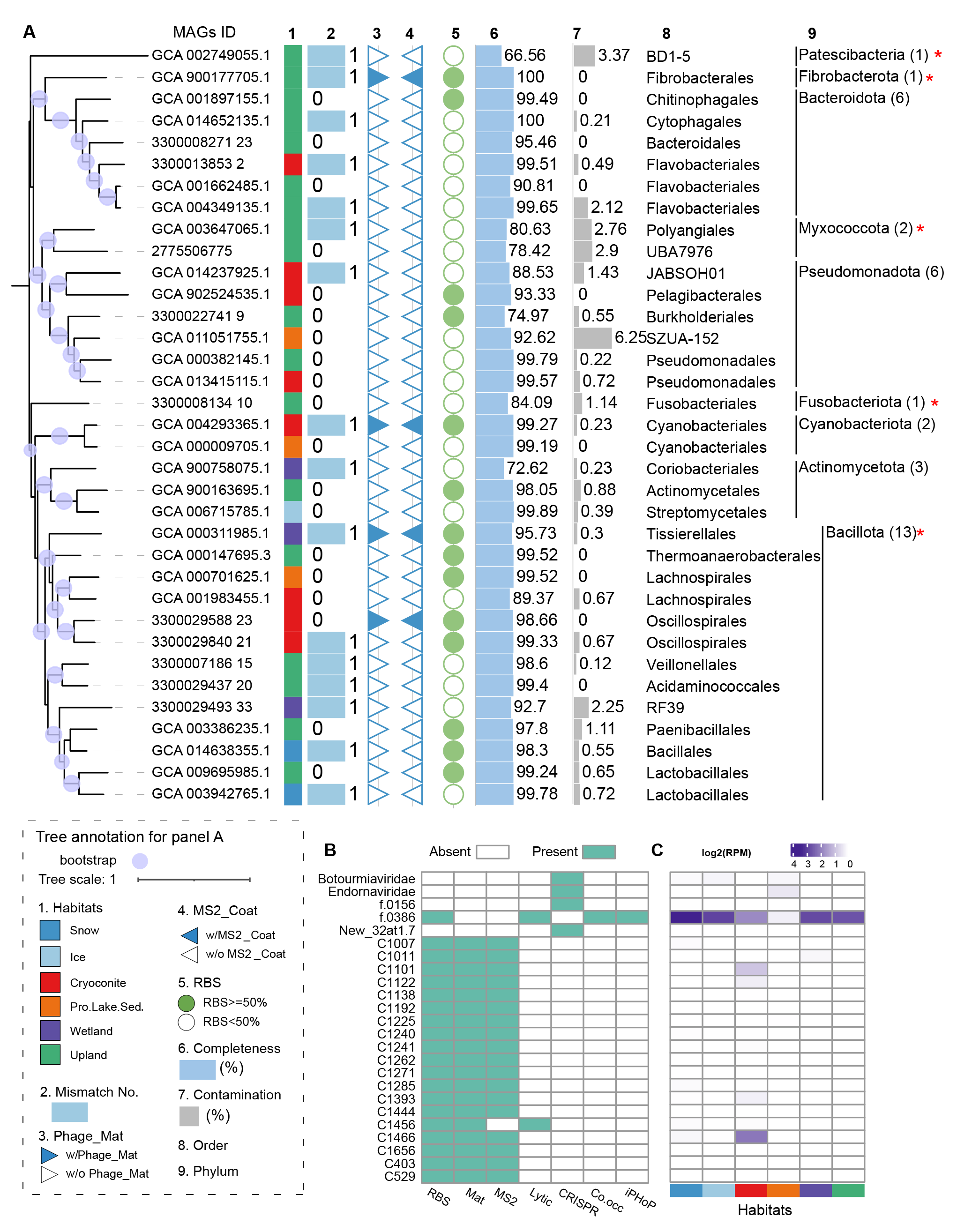


Fig. S10. Extended prokaryotic hosts for RNA viruses.

(**A**) Phylogenomic tree of Bacteria with CRISPR-spacer linkages to vContigs. The tree annotations from the left to the right are: 1, color pallets show the sources of vContigs (e.g., cryoconite); 2, bars show the number of mismatches between vContigs and the CRISPR spacer of corresponding MAGs; 3, outwards triangles show whether vContigs coding phage MS2 coat gene (blue filled) or not (empty); 4, inwards triangles show whether vContigs coding phage maturation gene (Phage_Mat, blue filled) or not (empty); 5, dots show whether vContigs with RBS ratio ≥50% (filled green) or not (empty); 6, the completeness of the MAGs; 7, contamination of the MAGs; 8 the Order of the MAGs and 9 the Phylum of the MAGs. One archaeal phylum (Halobacteriota) with RNA virus linkage is not shown here. Bootstrap values >75% are depicted by dots on the tree. The number of MAGs is shown after the phylum name. Phylum with red asterisk (*) are not recognized as RNA virus hosts in previous studies. (**B**) Summary of extended prokaryotic RNA virus signals for established RNA viral families and unclassified RNA virus clades. (**C**) Heatmap showing the relative abundance of extended prokaryotic RNA viruses. The colour code for habitats in C is the same as in panel A. Panel B shared the same row label as C. In panel C, only groups with ≥ 3 putative prokaryotic vContigs are shown. See Table S5 for more information on other putative prokaryotic vContigs. The RPM is the summary of all putative prokaryotic vContigs belonging to that group (e.g., f.0386). Abbreviations: Mat, Phage maturation protein; MS2, phage MS2 coat protein; Lytic, phage lytic protein; CRISPR, CRISPR spacer linkage; co-occ, co-occurrence; w, with; w/o, without.


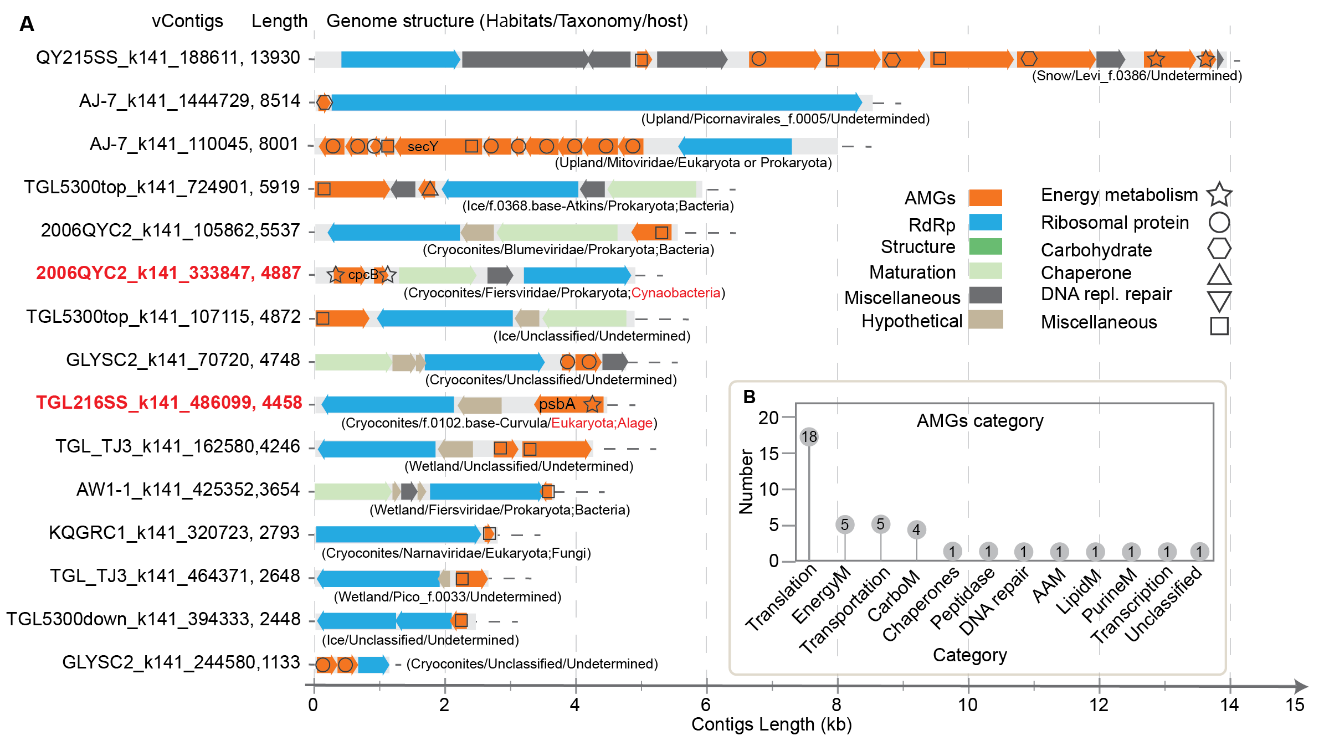


Fig. S11. Genome structure of vContigs with AMGs and number of AMGs belonging to different functional categories.

**(A)** Genome structure of vContigs with AMGs. The two vContigs encoding *cpcB* and *psbA* are shown in red. **(B)** Number of AMGs belonging to different functional categories.


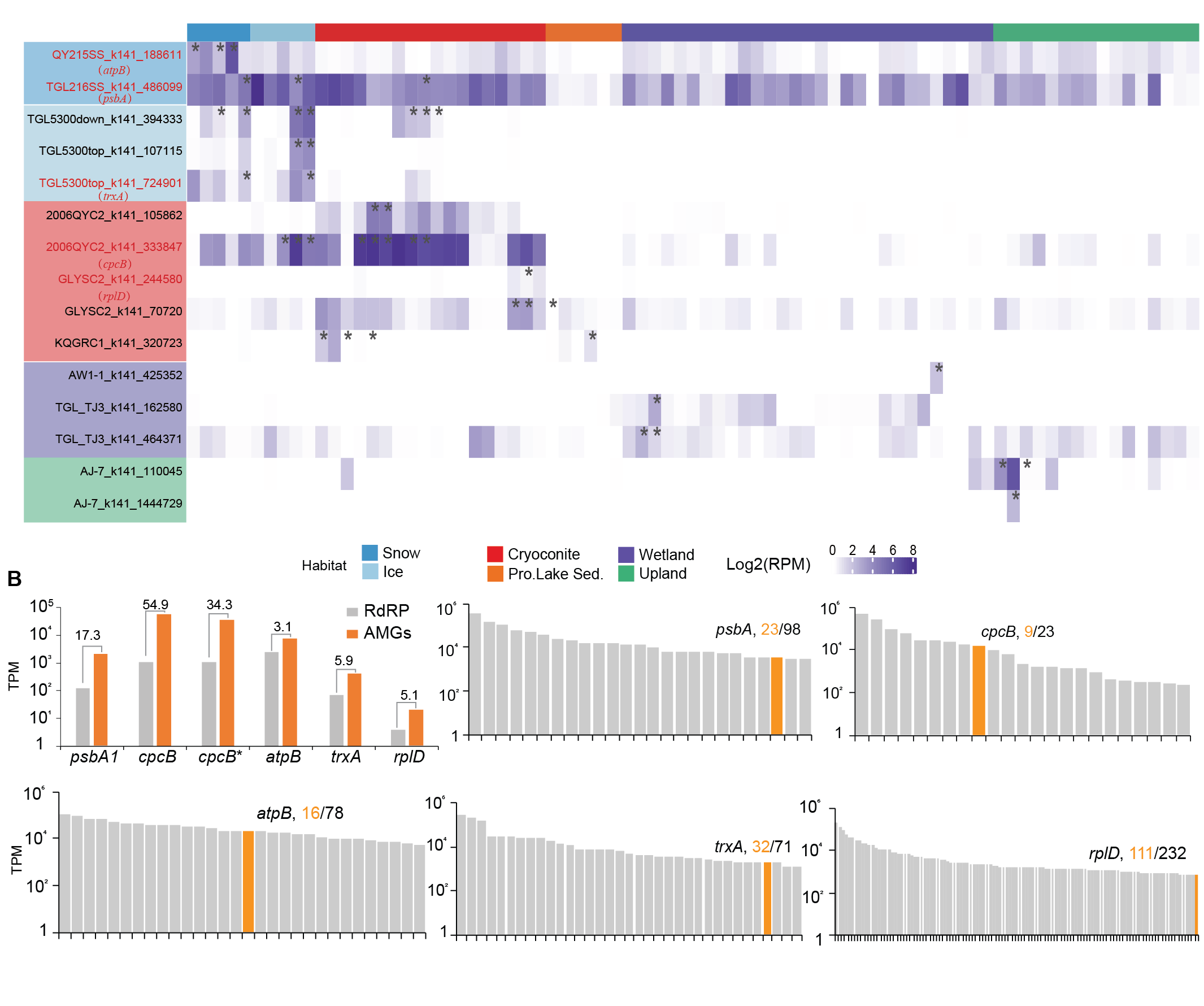


Fig. S12. AMGs of the TPC RNA viruses.

(**A**) The heatmap showing the RPM and reads coverage of RNA viruses with AMGs across different samples. Row labels are vContigs names, with those harboring 5 selected AMGs highlighted in red. Selected gene names are also shown alongside the vContigs. Asterisks indicate vContigs with horizontal coverage >80% in corresponding samples. (**B**) TPM of RdRps coding genes, AMGs, and comparison of transcripts abundances of AMGs to non-viral encoded functional genes in corresponding samples of with the selected vContigs derived. The *psbA* encodes the photosystem II core reaction center protein D1 (TGL216SS_k141_486099). The *cpcB* encodes the phycocyanin protein beta chain (2006QYC2_k141_333847). The *trxA* encodes the thioredoxin protein (TGL5300top_k141_724901). The *atpB* gene encoded ATPase F0a protein in the snow RNA vContig (QY215SS_k141_188611). The *rplD* encodes the large subunit ribosomal protein L4, which plays a key role in protein synthesis (GLYSC2_k141_244580). The vContig (2006QYC2_k141_333847) harboring *cpcB* gene has two CpcB functional domains, * indicate the second CpcB domain. The horizontal axis of the following charts represents the number of functional genes (e.g., *psbA*) identified from the metatranscriptome-assembled contigs in the sample of which the AMG was obtained. Additionally, the TPM rank of the AMG relative to the total number of functional genes identified is indicated in the upper right corner of each figure as #/# (e.g., TPM rank of the AMG/ TPM rank of all functional genes). All these selected genes showed high transcript abundances in the community.


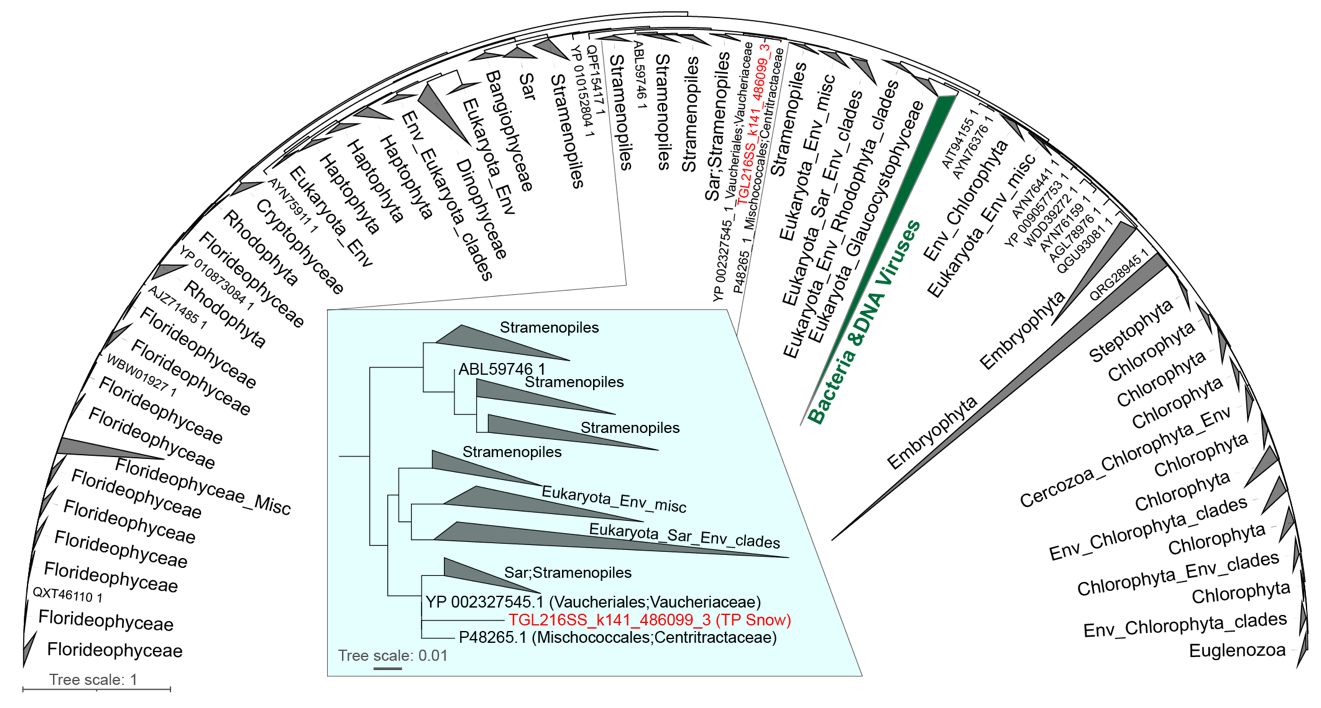
Fig. S13. Maximum-likelihood tree of the protein sequences of PsbA1.

PsbA1 from the vContigs is highlighted in red and the sub-clade with this sequence is shown in the inserted panel. The clade of PsbA1 protein encoded by Bacteria and DNA viruses is colored in deep green. The RNA viruses coded by vContigs from TP snow are closely related to Stramenopiles (Sar supergroup), Vaucheriaceae (a family in Chromista, brown algae and allies, YP_002327545.1) and Centritractaceae (a family in Xanthophyceae, yellow-green algae, P48265.1). The maximum-likelihood tree was constructed by using the FastTree software with the parameters “-gamma -lg -boot 1000”. Reference sequences were obtained from NCBI with IPG database by searching with “photosystem II protein D1” and filtered out none PsbA1 domain using hmmsearch with TIGR01151.1.hmm and PF00124.hmm. The gathering score of 27 and length of 180 aa was used for filtering. After filtering, all reference PsbA1 aa sequences were dereplicated using CD-HIT with 0.99 as the cutoff. PsbA1 and non-redundant reference PsbA1 were combined for muscle alignment, trimal (with -gappyout) and gap removal (drop sequences with >70% gaps). The final multiple sequence alignments were used for tree construction.

**
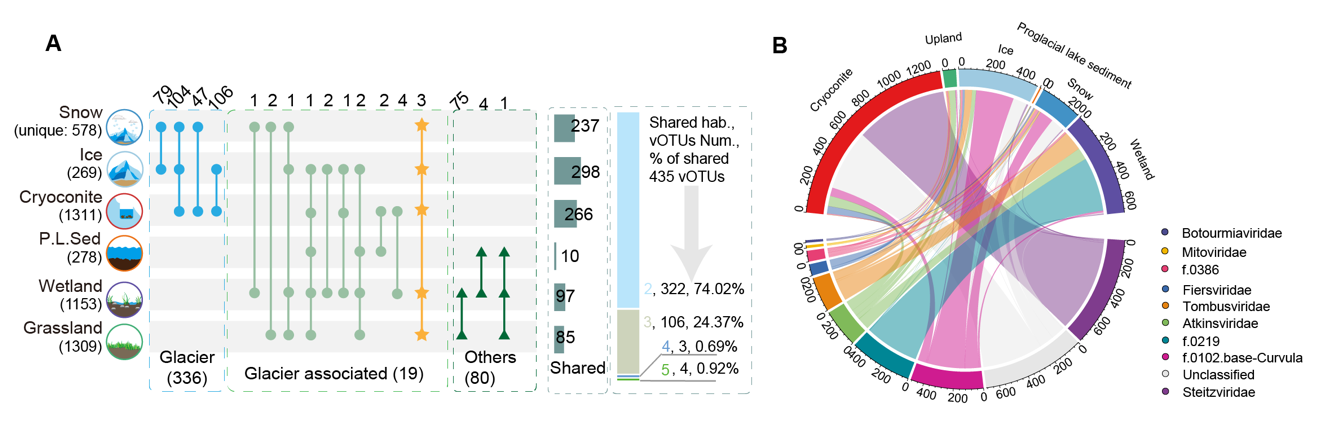
**

Fig. S14. Shared number of vOTUs across different habitats.

**(A)** Upset plot showing the number of shared and unique RNA vOTUs across Ecosystems and Habitats. **(B)** median RPM of shared vOTUs. The RPM of those shared vOTUs was grouped at the family levels.

**
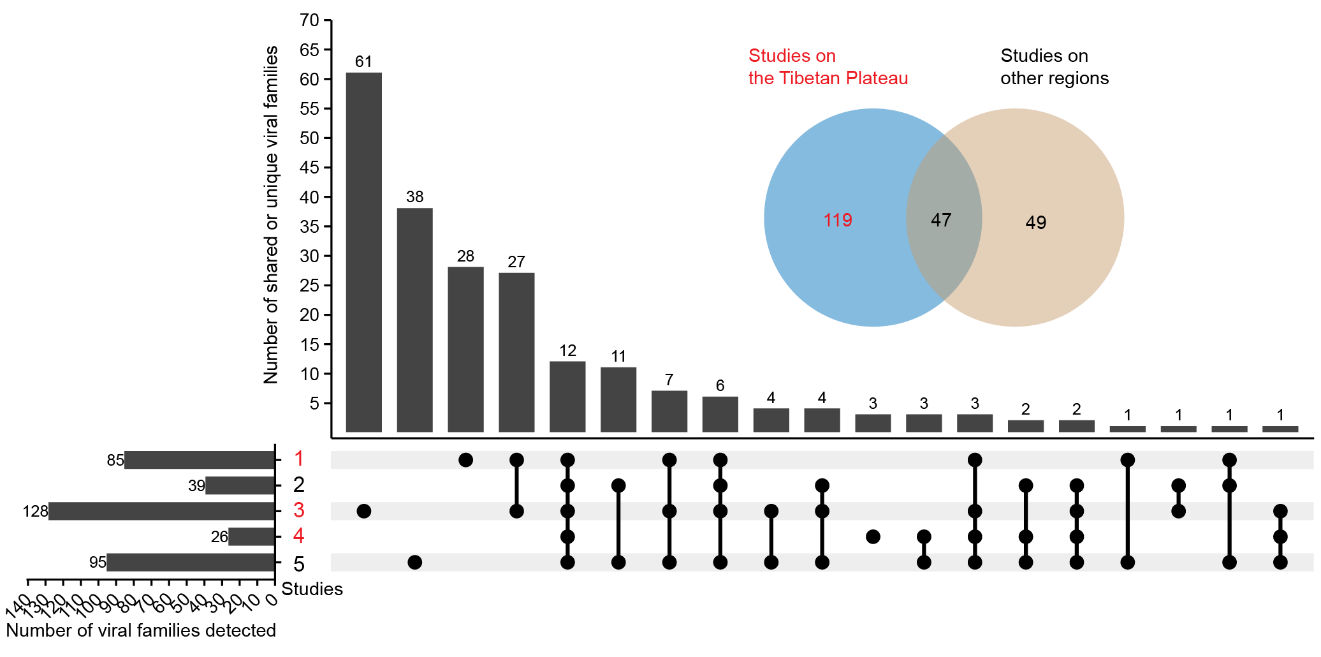
**

Fig. S15. Comparison of detected RNA virus families between our data and other cryosphere studies.

Each number on the y-axis of the lower part depicted studies we compared. 1, Wu et al., 2025 (doi:10.1073/pnas.2420162122), the study of the Nam Co lake water RNA virome on the Tibetan Plateau; 2, Wu et al., 2022 (doi:10.1128/msystems.00582-22), the study of thawed Alaska permafrost soil RNA virome; 3, current study; 4, Wen et al., 2024 (doi:10.1016/j.scitotenv.2024.172829), the study of the permafrost soil RNA virome from the surface to the depth up to 100 meters; 5, Pratama et al., 2025 (doi:10.1101/2025.02.13.637936), the study of the Stordalen Mire soil RNA virome. Studies from the Tibetan Plateau are shown in red. The inset Venn diagram illustrates the number of shared and unique viral families between studies conducted on the Tibetan Plateau (studies 1, 3, and 4) and those from other regions (studies 2 and 5). Unique and shared families between studies can be found in Table S10.


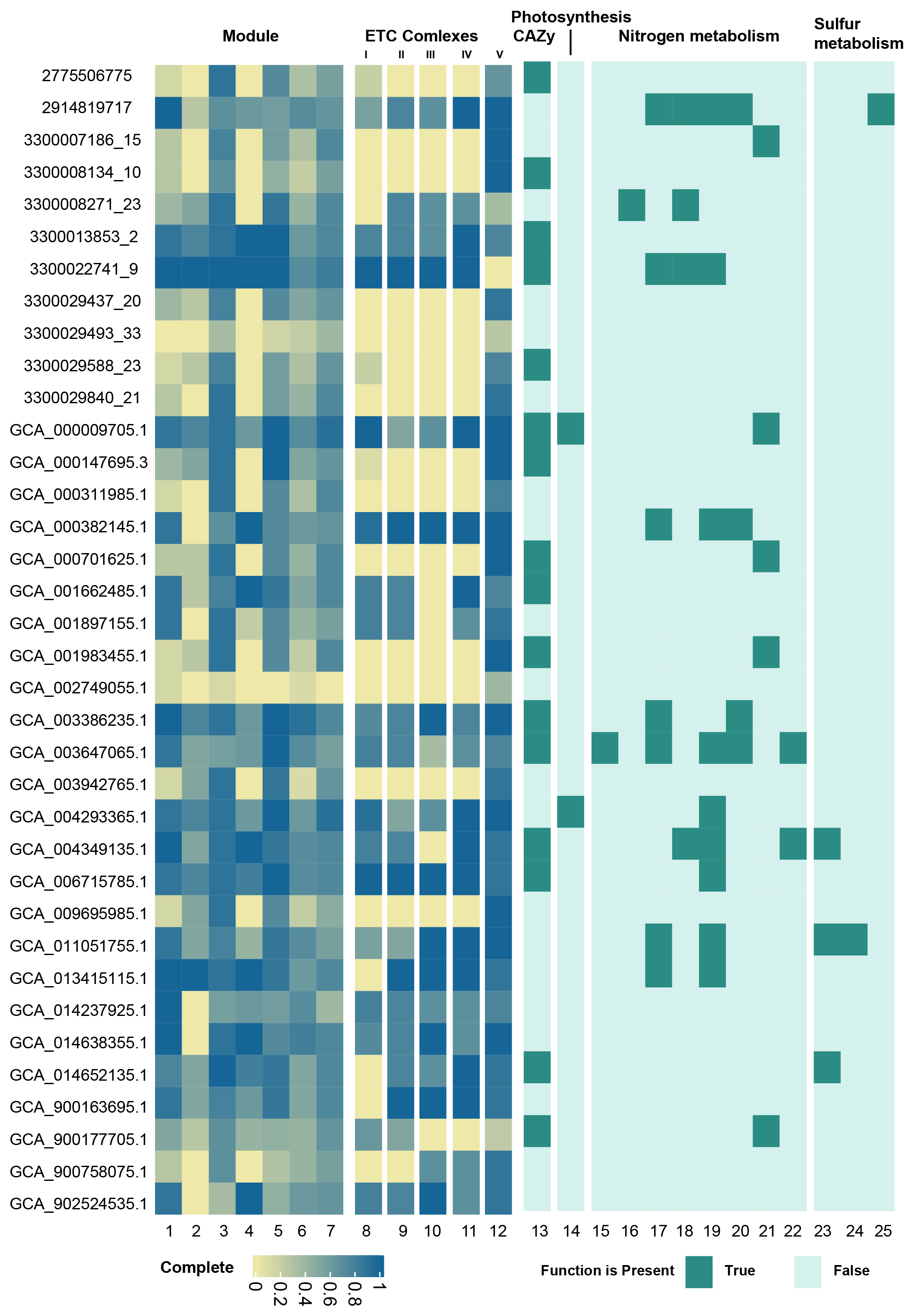


Fig. S16. Genome annotation of MAGs with at least one CRISPR-linkage to RNA vContigs.

The number on the bottom of the heatmap depicted the pathways.

Central pathway: 1, Citrate cycle (TCA cycle, Krebs cycle); 2, Entner-Doudoroff pathway (glucose-6P => glyceraldehyde-3P + pyruvate); 3, Glycolysis (Embden-Meyerhof pathway, glucose => pyruvate); 4, Glyoxylate cycle; 5, Pentose phosphate pathway (Pentose phosphate cycle); 6, Reductive citrate cycle (Arnon-Buchanan cycle); 7, Reductive pentose phosphate cycle (Calvin cycle). The membrane-bound respiratory chain: 8, Complex I; 9, Complex II; 10, Complex III; 11, Complex IV; 12, Complex V; Element cycling and energy metabolism: 13, CAZY; 14, Photosynthesis: Photosystem; 15, Aerobic-specific ammonia oxidation; 16, Dissimilatory nitrite reduction to ammonia (DNRA); 17, Nitrate reduction (nitrate => nitrite); 18, Nitric oxide => nitrous oxide; 19, Nitrite => nitric oxide; 20, Nitrite=> nitrate; 21, Nitrogen => ammonia; 22, Nitrous oxide => nitrogen; 23, Thiosulfate oxidation by SOX complex, (thiosulfate => sulfate); 24, Dissimilatory sulfate reduction (and oxidation) sulfate => sulfide; 25, Tetrathionate => thiosulfate.


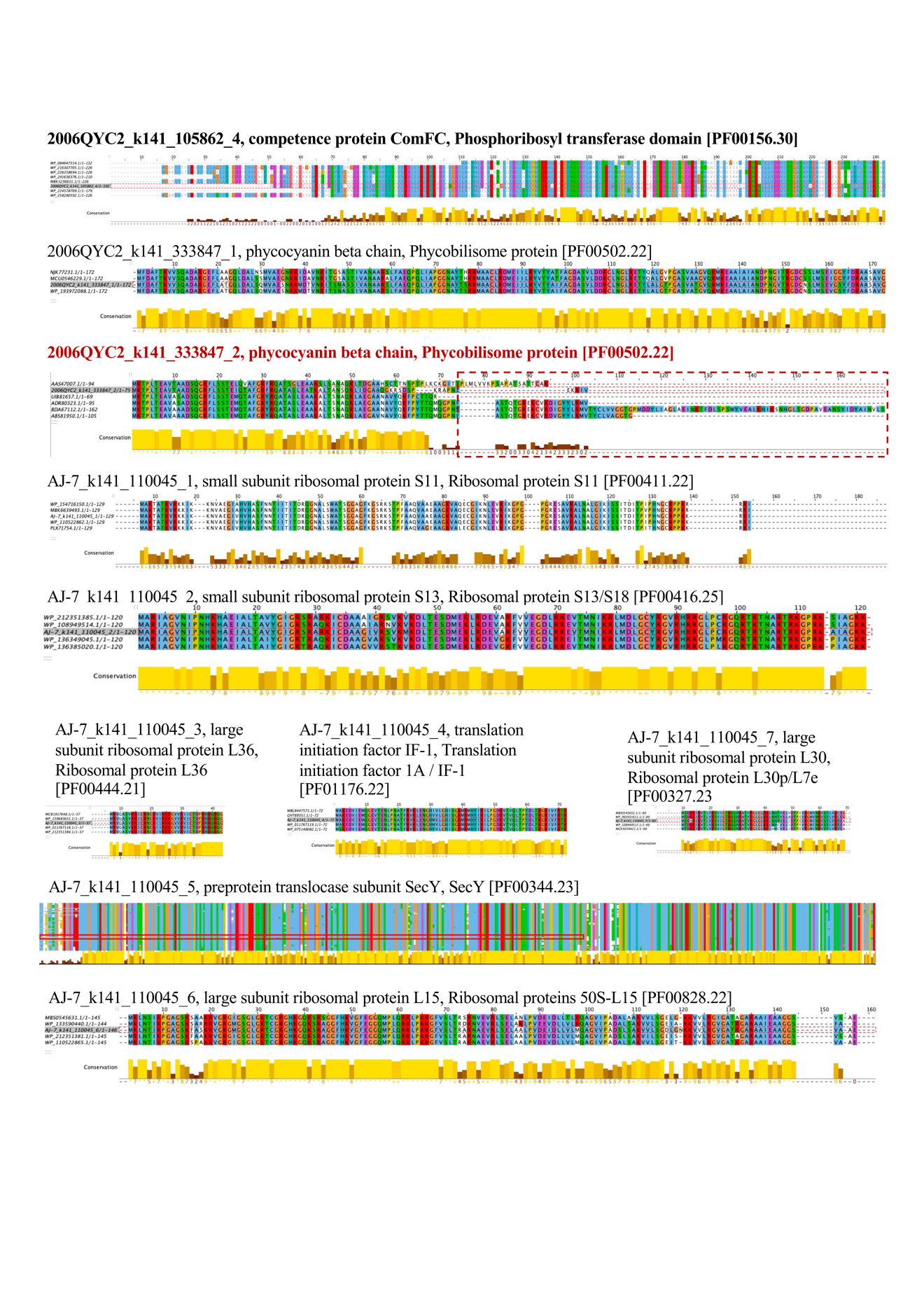


Fig. S17. Sequences alignments of AMGs amino acid sequences with their closest cellular homologs in NR database.

The sequences with names covered by grey on the left side were the amino acid sequences of AMG while others were reference sequences retrieved from NCBI by Blastp. The conservation status of residues was shown on the bottom of each alignment. AMG amino acid sequences that are partial of the full ORF compared to the references are highlighted in red. The missing parts within the alignment were also highlighted with bold red rectangles. The description is shown beside the gene name. See the following pages for other AMGs and Supplementary results and discussions for more details. Due to limited space for each alignment**,** we also upload the full multiple sequences alignment files for each AMG to figshare (https://doi.org/10.6084/m9.figshare.c.7390684.)

**
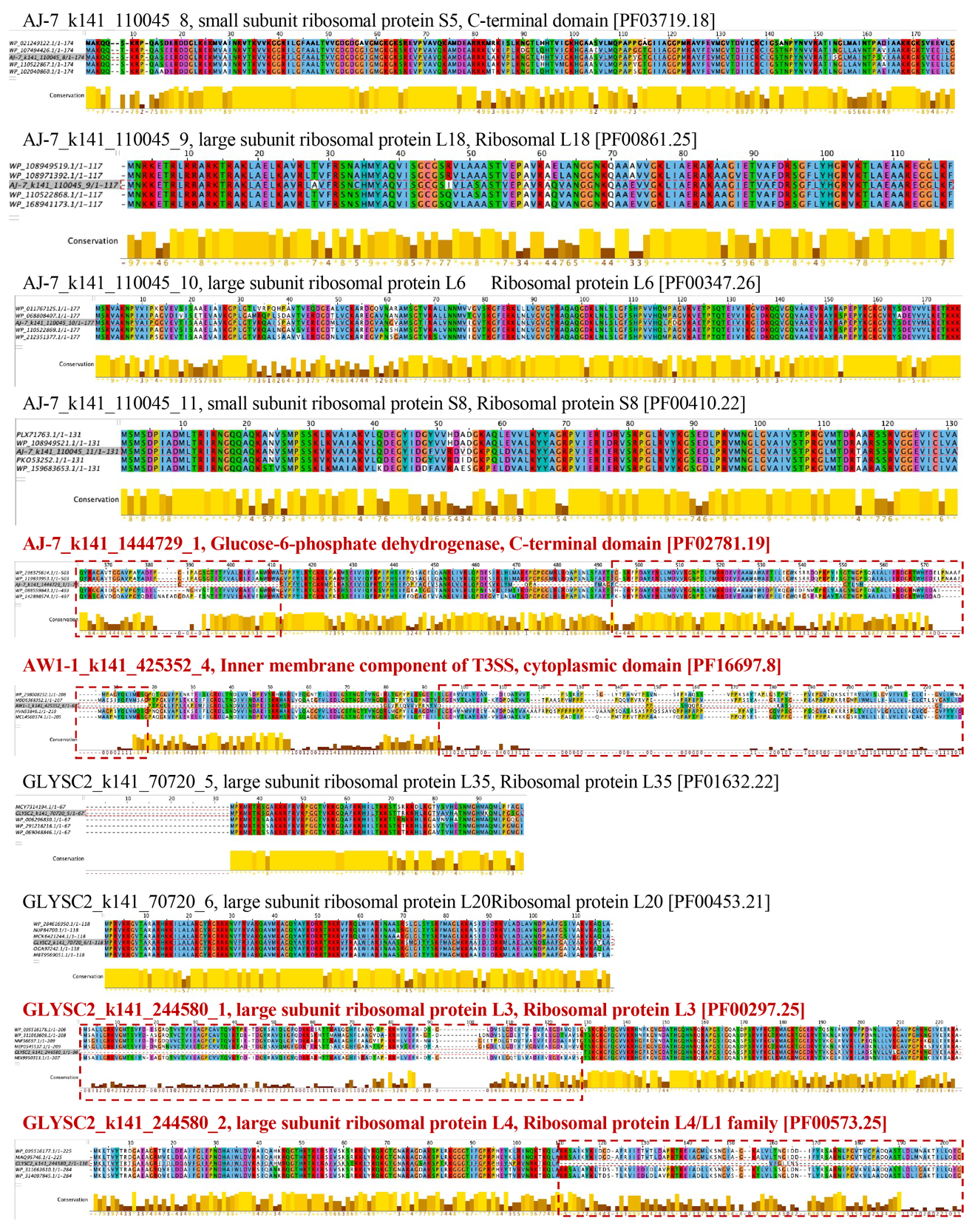
**

Fig. S17. Sequences alignments of AMGs amino acid sequences with their closest cellular homologs in NR database. Continue.

**
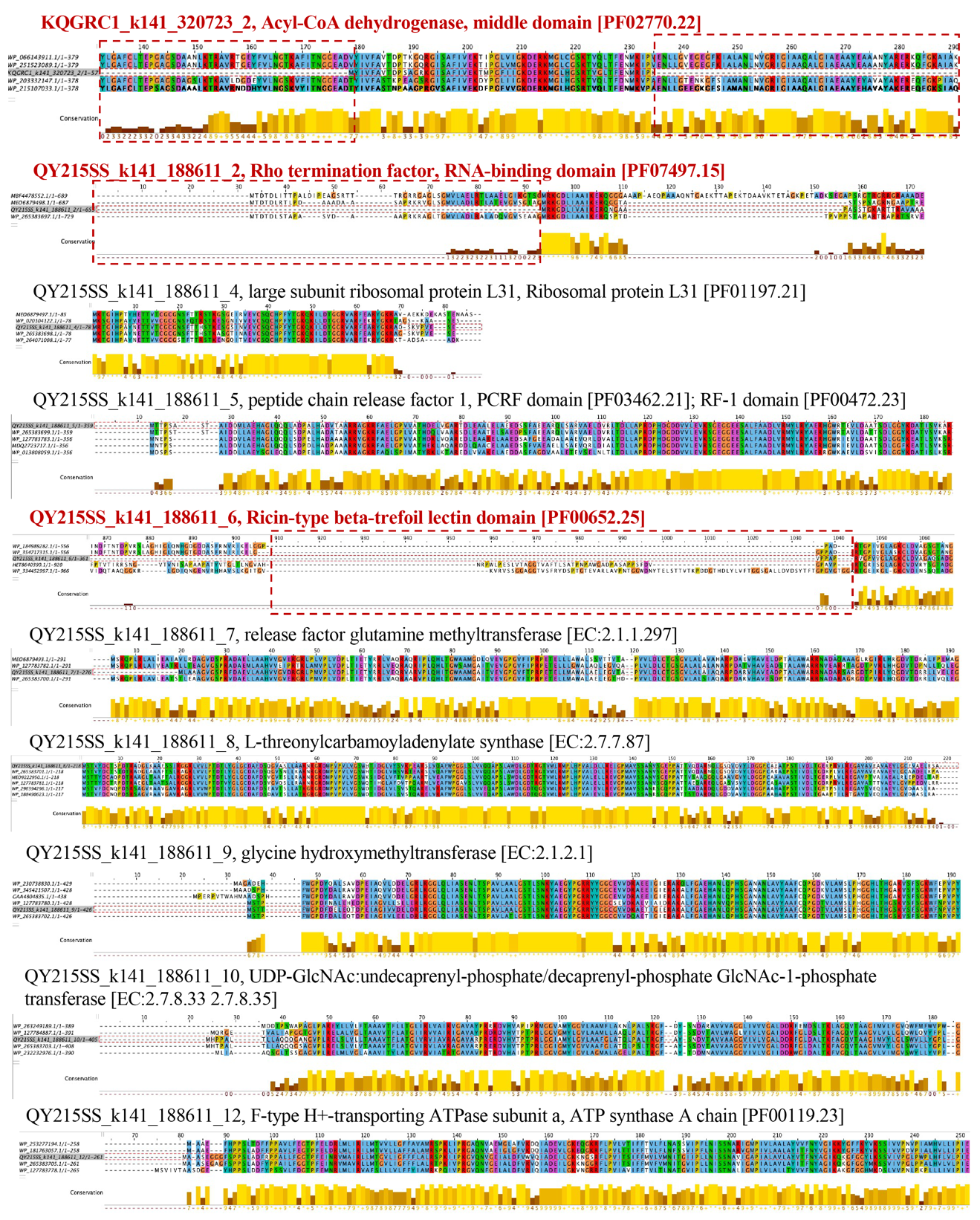
**

Fig. S17. Sequences alignments of putative AMGs amino acid sequences with their closest cellular homologs found in NR database. Continue.

**
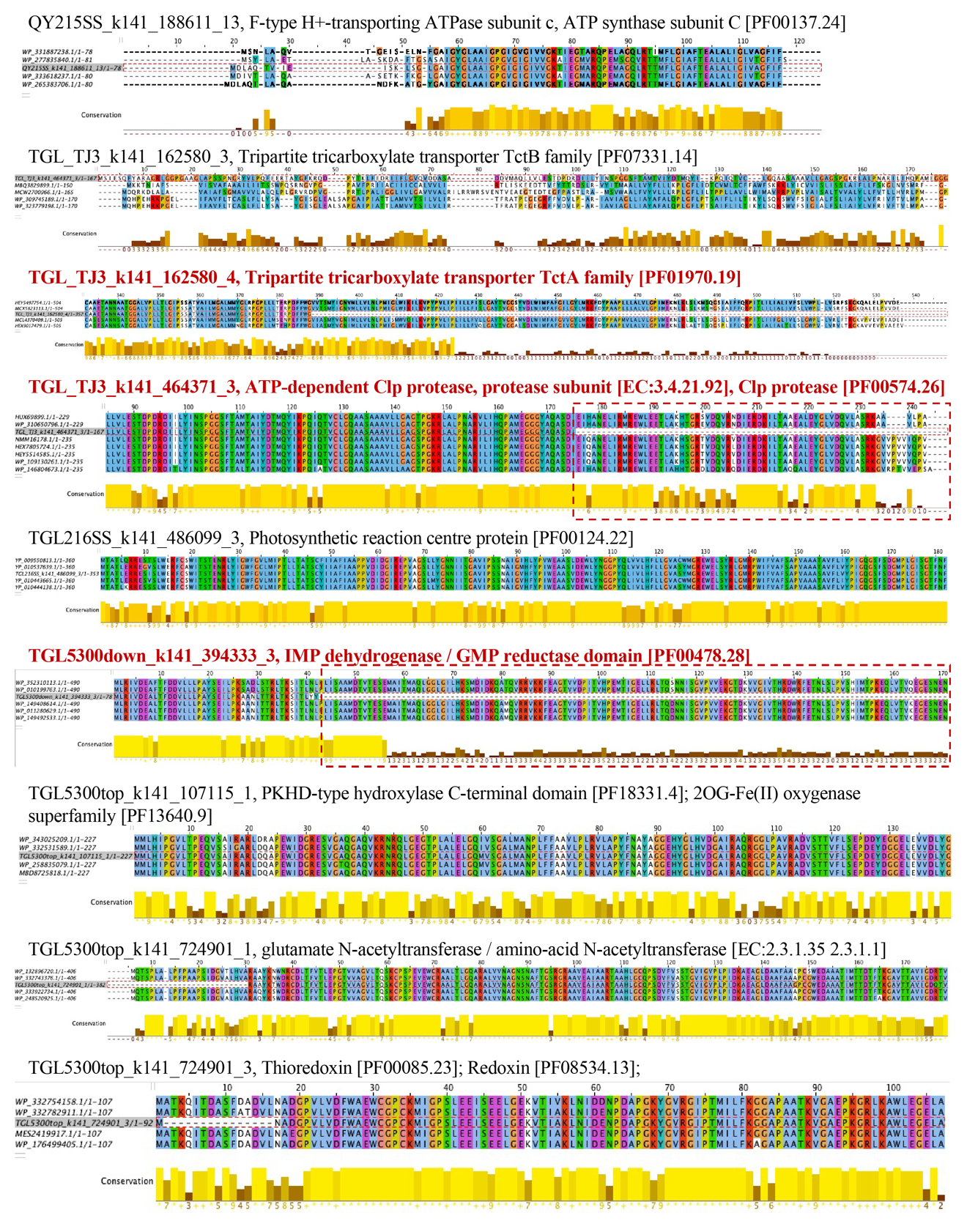
**

Fig. S17. Sequences alignments of putative AMGs amino acid sequences with their closest cellular homologs found in NR database. Continue.


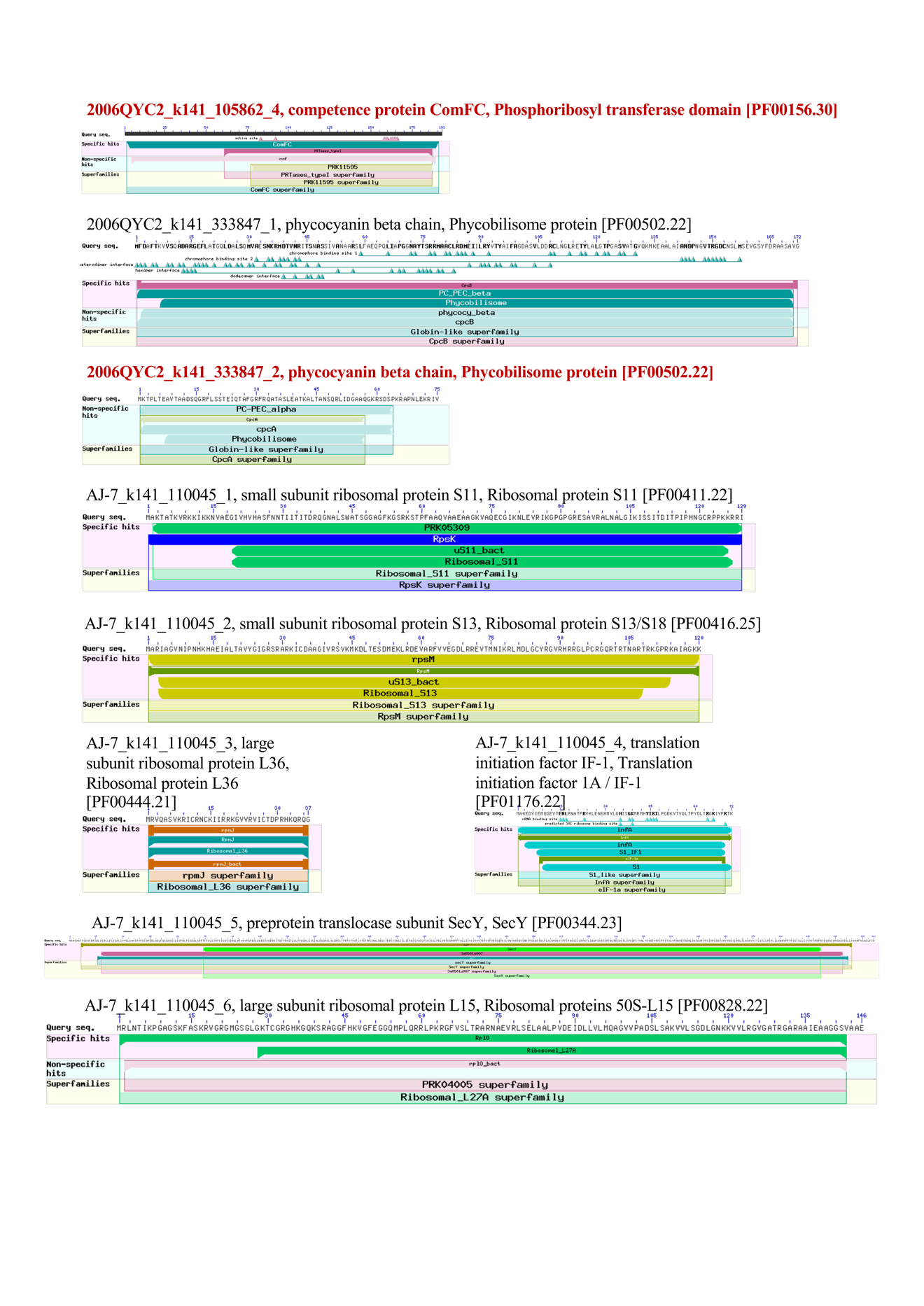


Fig. S18. Domain analysis of putative AMGs.

AMG amino acid sequences were searched against the (<https://www.ncbi.nlm.nih.gov/Structure/cdd/wrpsb.cgi>) for detailed functional domain annotation to verify the function of each AMG. The description is shown beside the gene name. See the following pages for other AMGs and Supplementary results and discussions for more details. Due to limited space, a PowerPoint file with high-resolution figures (together with Fig. S17) was also uploaded to figshare (https://doi.org/10.6084/m9.figshare.c.7390684


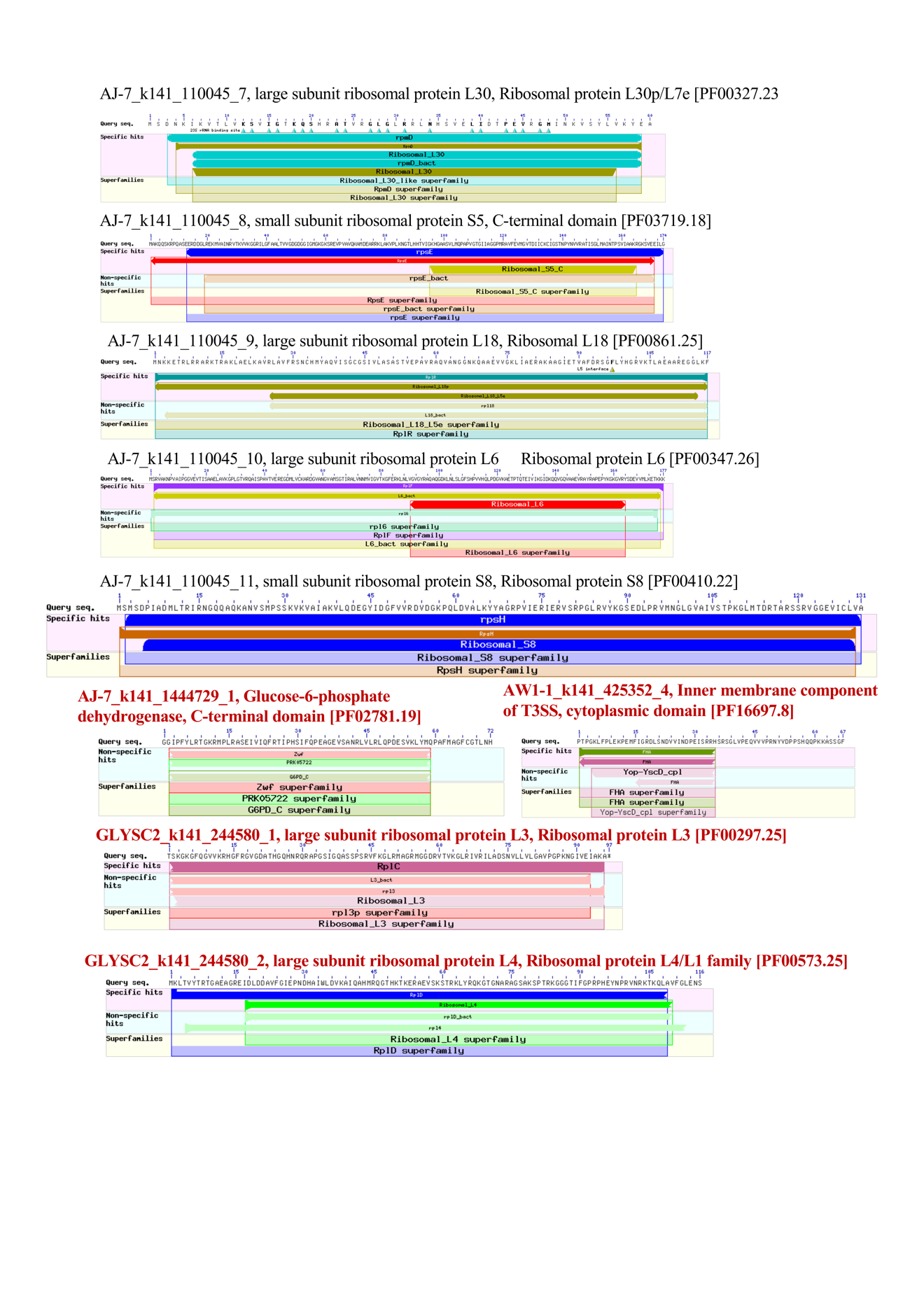


Fig. S18. Domain analysis of putative AMGs. Continue.

**
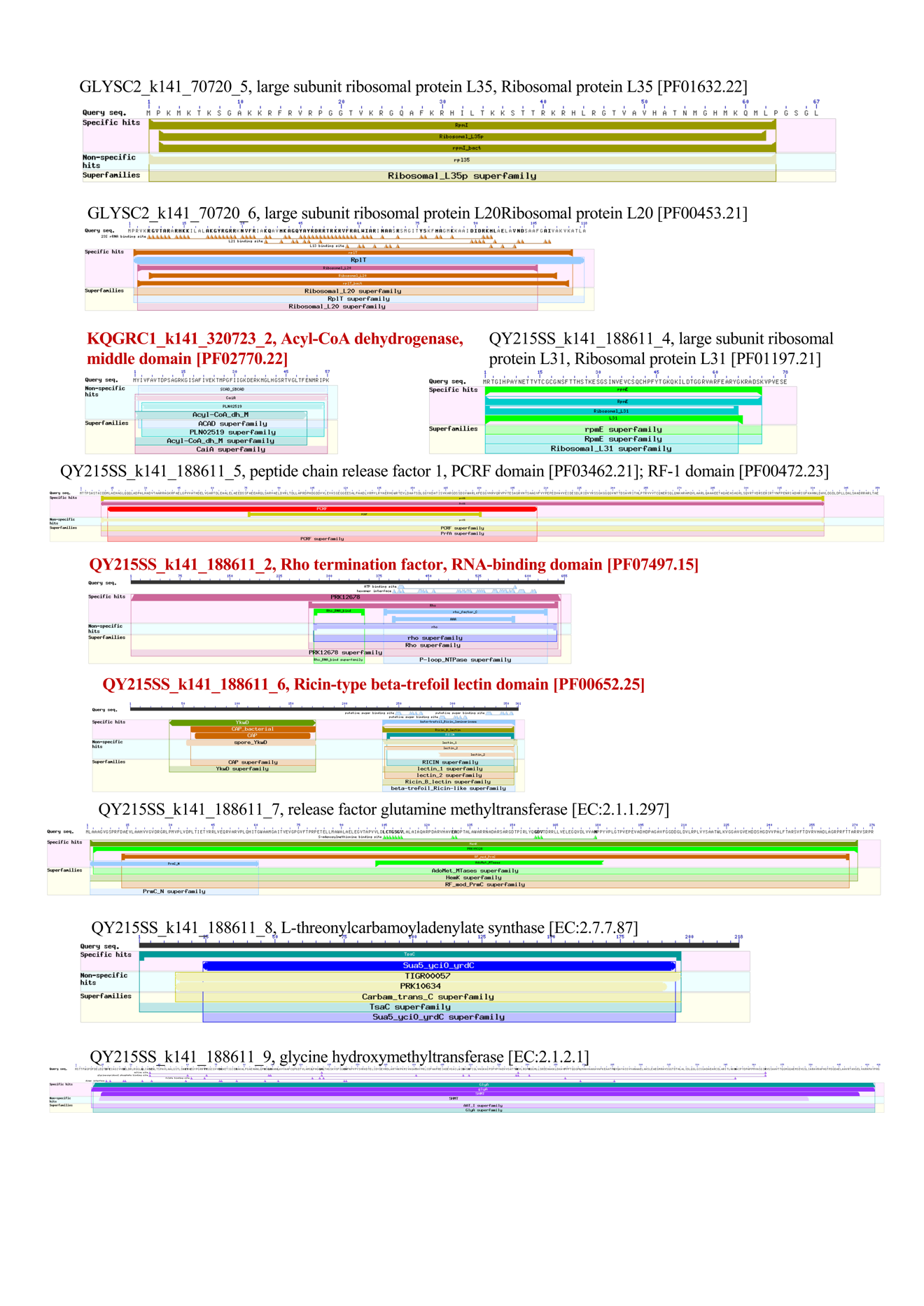
**

Fig. S18. Domain analysis of putative AMGs. Continue.

**
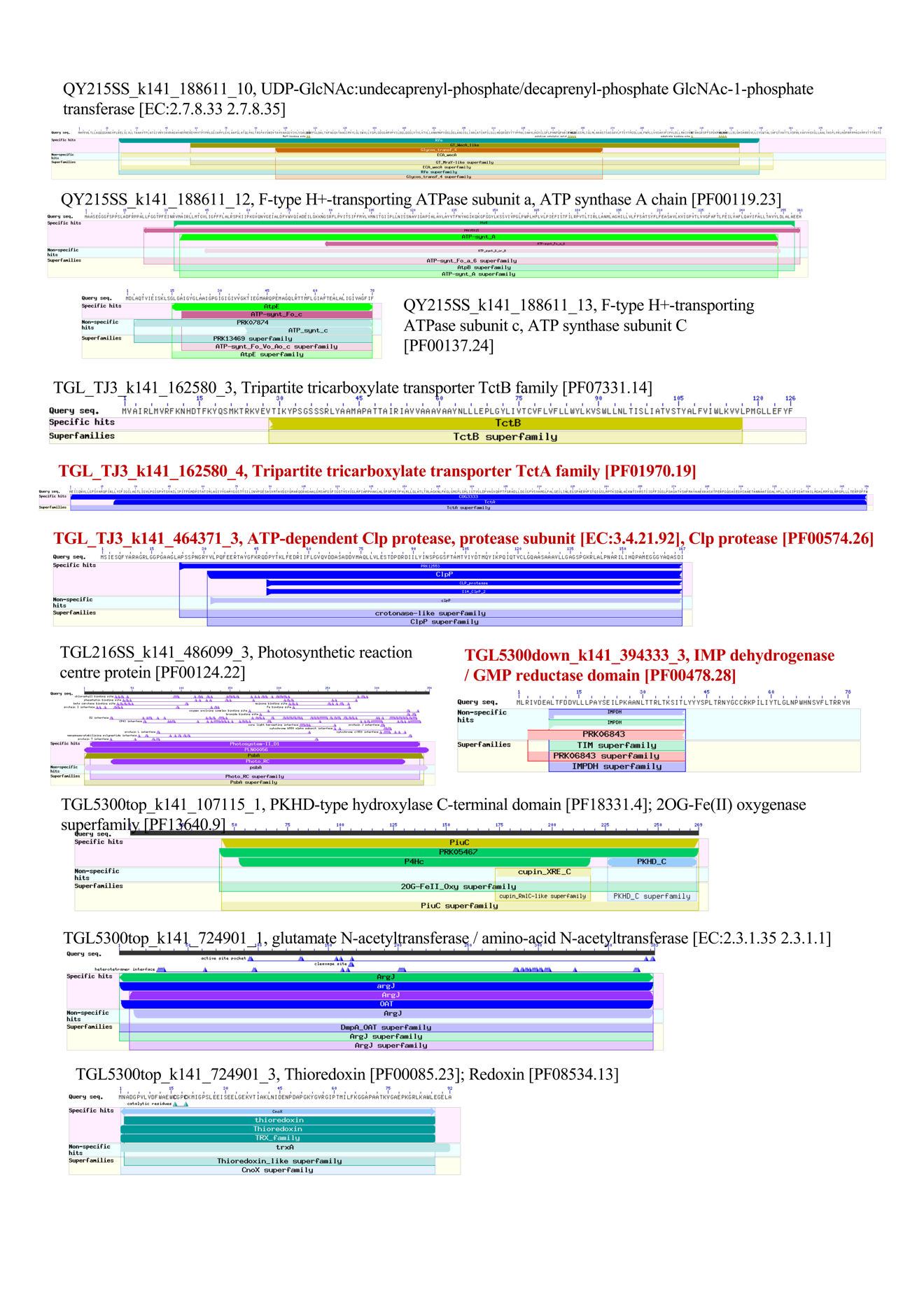
**

Fig. S18. Domain analysis of putative AMGs.

Continue.


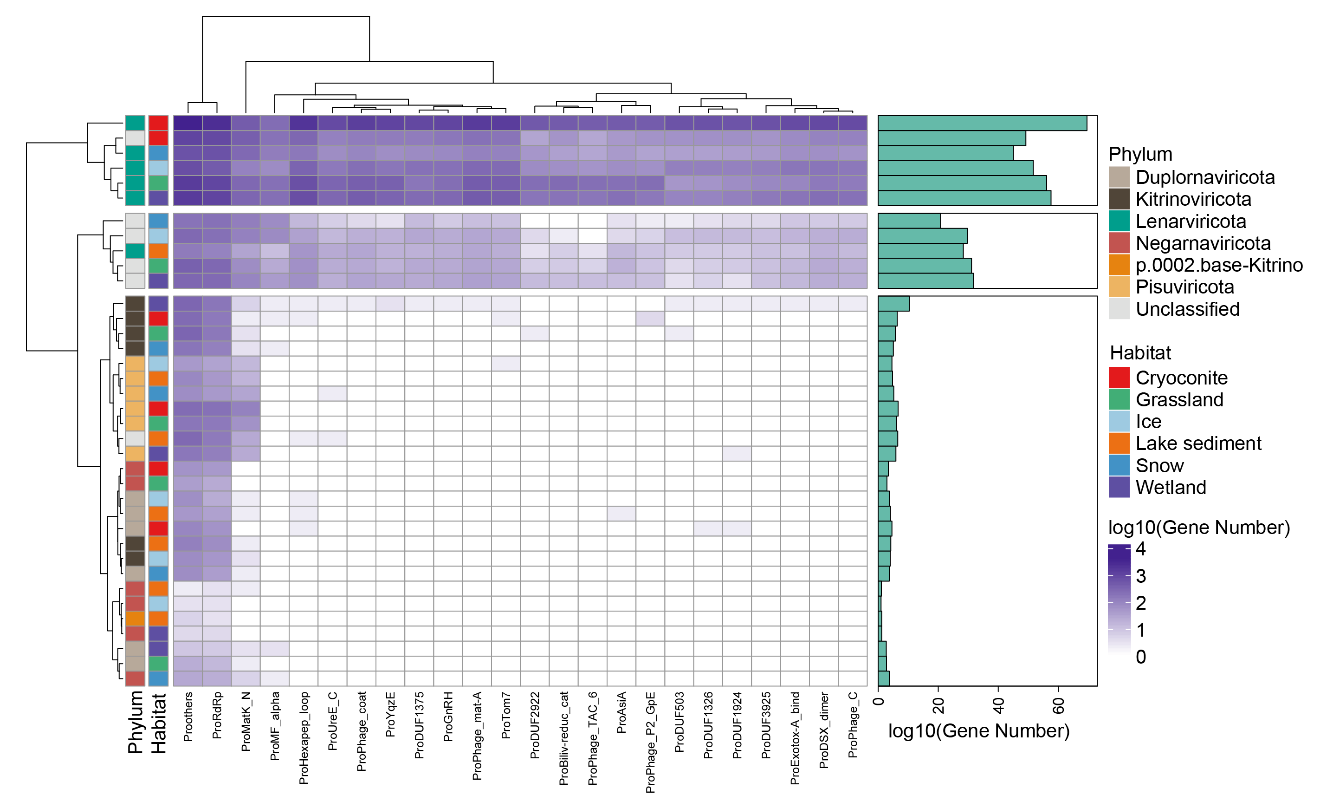


Fig. S19. Functional profile of TPC RNA viral genomes.

Rows represent RNA viral phylum from the corresponding habitat as indicated by the legends on the right. The columns of the heatmap represent the functional domains of RNA viral proteins. The color of each cell represents log10 transformed the number of proteins belonging to the phylum of that habitat. The green bars on the right represent the total number of functional domains for the corresponding rows.


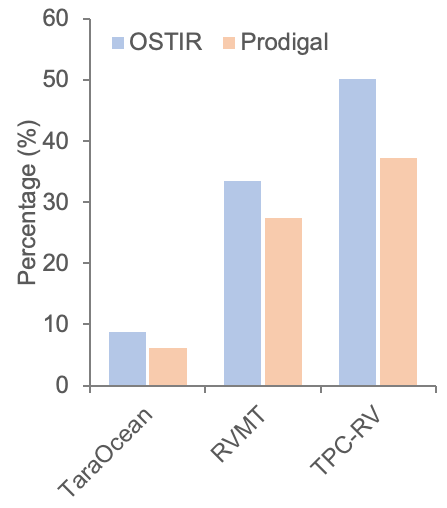


Fig. S20. Percentage of RNA viral genomes with RBS ratio ≥ 50%.

Two pipelines were used to identify the RBS motifs within each vContigs, including OSTIR (Roots et al., 2021, J Open Source Softw, 10.21105/joss.03362) and prodigal (Hyatt et al., 2010, BMC Bioinformatics, 10.1186/1471-2105-11-119).


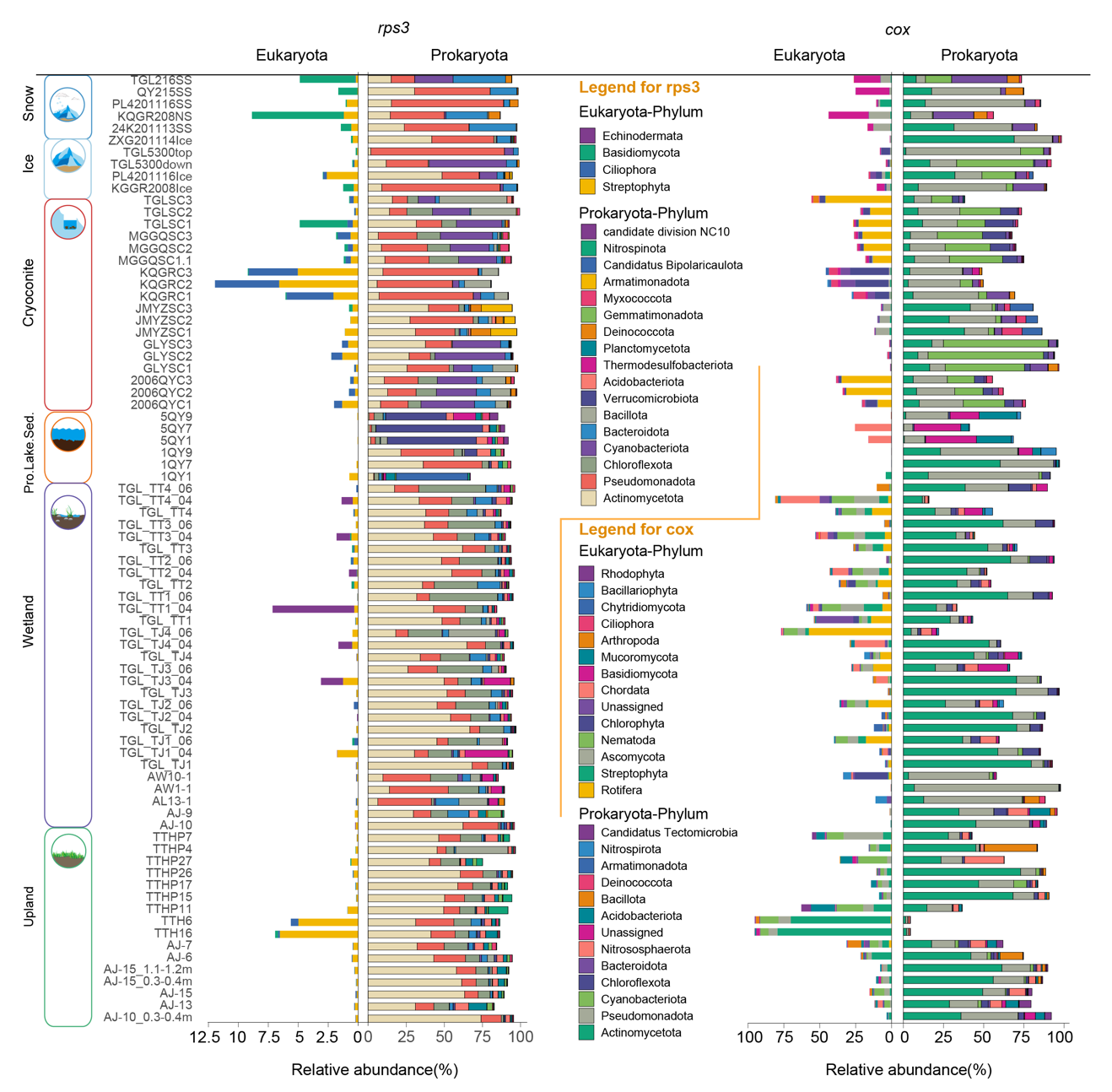


Fig. S21. The relative abundance of *rps3* gene and *cox1* gene transcripts across habitats and samples.

Supplementary Tables S1-S12 (in a separate Excel file, available in figshare: https://doi.org/10.6084/m9.figshare.c.7390684).

Table S1 Metadata of metatranscriptomic samples and libraries.

Table S2 Genome features of RNA vContigs.

Table S3 Consensus taxonomic assignment of vContigs.

Table S4 RNA viral families and their corresponding hosts.

Table S5 Evidence for extended prokaryotic RNA viruses.

Table S6 CRISPR-spacer linkages between RNA viruses and prokaryotic hosts.

Table S7 Zoonotic risk prediction by Zoonotic rank.

Table S8 TPC RNA viruses encoded AMGs.

Table S9 BLASTp results of TPC RNA viruses encoded AMGs.

Table S10 Comparison of the RNA virome between the TPC and other cryosphere environments.

Table S11 Metagenome sample metadata.

Table S12 BLASTp results of unclassified TPC RNA viruses.

SI References

1. Hodson A, Anesio AM, Tranter M *et al.* Glacier ecosystems. *Ecol Monogr*. 2008; **78**(1): 41-67. doi: <https://doi.org/10.1890/07-0187.1>

2. Stibal M, Šabacká M, Žárský J. Biological processes on glacier and ice sheet surfaces. *Nat Geosci*. 2012; **5**(11): 771-774. doi: <https://doi.org/10.1038/ngeo1611>

3. Anesio AM, Lutz S, Chrismas NAM *et al.* The microbiome of glaciers and ice sheets. *NPJ Biofilms Microbiomes*. 2017; **3**(1): 10. doi: 10.1038/s41522-017-0019-0

4. Carrivick JL, Tweed FS. Proglacial lakes: character, behaviour and geological importance. *Quat Sci Rev*. 2013; **78**: 34-52. doi: <https://doi.org/10.1016/j.quascirev.2013.07.028>

5. Liu K, Liu Y, Han B-P *et al.* Bacterial community changes in a glacial-fed Tibetan lake are correlated with glacial melting. *Sci Total Environ*. 2019; **651**: 2059-2067. doi: <https://doi.org/10.1016/j.scitotenv.2018.10.104>

6. Liu K, Liu Y, Yan Q *et al.* Temperature-driven shifts in bacterioplankton biodiversity: Implications for cold-preferred species in warming Tibetan proglacial lakes. *Water Res*. 2024; **265**: 122263. doi: <https://doi.org/10.1016/j.watres.2024.122263>

7. Jiang H, Zhang W, Yi Y *et al.* The impacts of soil freeze/thaw dynamics on soil water transfer and spring phenology in the Tibetan Plateau. *Arct Antarct Alp Res*. 2018; **50**(1): e1439155. doi: 10.1080/15230430.2018.1439155

8. Yun H, Ciais P, Zhu Q *et al.* Changes in above- versus belowground biomass distribution in permafrost regions in response to climate warming. *Proc Natl Acad Sci U S A*. 2024; **121**(25): e2314036121. doi: 10.1073/pnas.2314036121

9. Wu M-H, Xue K, Wei P-J *et al.* Soil microbial distribution and assembly are related to vegetation biomass in the alpine permafrost regions of the Qinghai-Tibet Plateau. *Sci Total Environ*. 2022; **834**: 155259. doi: <https://doi.org/10.1016/j.scitotenv.2022.155259>

10. Bourquin M, Busi SB, Fodelianakis S *et al.* The microbiome of cryospheric ecosystems. *Nat Commun*. 2022; **13**(1). doi: 10.1038/s41467-022-30816-4

11. Zhao K, Jing X, Sanders NJ *et al.* On the controls of abundance for soil-dwelling organisms on the Tibetan Plateau. *Ecosphere*. 2017; **8**(7): e01901. doi: <https://doi.org/10.1002/ecs2.1901>

12. Wei X, Wu P. Responses of soil insect communities to alpine wetland degradation on the eastern Qinghai-Tibetan Plateau, China. *Eur J Soil Biol*. 2021; **103**: 103276. doi: <https://doi.org/10.1016/j.ejsobi.2020.103276>

13. Wei X, Cao R, Wu X *et al.* Effect of water table decline on the abundances of soil mites, springtails, and nematodes in the Zoige peatland of eastern Tibetan Plateau. *Appl Soil Ecol*. 2018; **129**: 77-83. doi: <https://doi.org/10.1016/j.apsoil.2018.05.006>

14. Chen Y-M, Sadiq S, Tian J-H *et al.* RNA viromes from terrestrial sites across China expand environmental viral diversity. *Nat Microbiol*. 2022. doi: 10.1038/s41564-022-01180-2

15. Wu R, Zimmerman Amy E, Hofmockel Kirsten S. The direct and indirect drivers shaping RNA viral communities in grassland soils. *mSystems*. 2024; **0**(0): e00099-00024. doi: 10.1128/msystems.00099-24

16. Wu R, Davison MR, Gao Y *et al.* Moisture modulates soil reservoirs of active DNA and RNA viruses. *Commun Biol*. 2021; **4**(1): 992. doi: 10.1038/s42003-021-02514-2

17. Wu R, Bottos EM, Danna VG *et al.* RNA viruses linked to eukaryotic hosts in thawed permafrost. *mSystems*. 2022; **7**(6): e00582-00522. doi: doi:10.1128/msystems.00582-22

18. Hillary LS, Adriaenssens EM, Jones DL *et al.* RNA-viromics reveals diverse communities of soil RNA viruses with the potential to affect grassland ecosystems across multiple trophic levels. *ISME commun*. 2022; **2**(1): 34. doi: 10.1038/s43705-022-00110-x

19. Dominguez-Huerta G, Zayed AA, Wainaina JM *et al.* Diversity and ecological footprint of Global Ocean RNA viruses. *Science*. 2022; **376**(6598): 1202-1208. doi: doi:10.1126/science.abn6358

20. Wen Q, Yin X, Moming A *et al.* Viral communities locked in high elevation permafrost up to 100 m in depth on the Tibetan Plateau. *Sci Total Environ*. 2024; **932**: 172829. doi: <https://doi.org/10.1016/j.scitotenv.2024.172829>

21. Xing T, Liu Y, Dong X *et al.* Glacier melting promotes methane emission via increased methanogenic activity in the foreland alpine meadow. *Sci Total Environ*. 2024; **955**: 176947. doi: <https://doi.org/10.1016/j.scitotenv.2024.176947>

22. Liu K, Liu Y, Jiao N *et al.* Bacterial community composition and diversity in Kalakuli, an alpine glacial-fed lake in Muztagh Ata of the westernmost Tibetan Plateau. *FEMS Microbiol Ecol*. 2017; **93**(7): fix085. doi: 10.1093/femsec/fix085

23. Yue L, Kong W, Li C *et al.* Dissolved inorganic carbon determines the abundance of microbial primary producers and primary production in Tibetan Plateau lakes. *FEMS Microbiol Ecol*. 2021; **97**(2): fiaa242. doi: 10.1093/femsec/fiaa242

24. Bouvy M, Bettarel Y, Bouvier C *et al.* Trophic interactions between viruses, bacteria and nanoflagellates under various nutrient conditions and simulated climate change. *Environ Microbiol*. 2011; **13**(7): 1842-1857. doi: 10.1111/j.1462-2920.2011.02498.x

25. Yao T, Thompson L, Yang W *et al.* Different glacier status with atmospheric circulations in Tibetan Plateau and surroundings. *Nat Clim Change*. 2012; **2**(9): 663-667. doi: 10.1038/nclimate1580

26. Mo Y, Li X, Xu M. Regional differences in glacier changes and responses to climate warming in Asia. *Res Cold Arid Reg*. 2024. doi: <https://doi.org/10.1016/j.rcar.2024.12.004>

27. Guo B, Liu Y, Liu P *et al.* Glacial snow and ice contribute differentially to the dissolved organic matter in the runoff of Qiangyong Glacier, Tibetan Plateau. *J Hydrol*. 2025; **652**: 132600. doi: <https://doi.org/10.1016/j.jhydrol.2024.132600>

28. Wu L, Liu Y, Shi W *et al.* Uncovering the hidden RNA virus diversity in Lake Nam Co: Evolutionary insights from an extreme high-altitude environment. *Proc Natl Acad Sci U S A*. 2025; **122**(6): e2420162122. doi: 10.1073/pnas.2420162122

29. Pratama AA, Domínguez-Huerta G, Wainaina JM *et al.* RNA virus ecogenomics along a subarctic permafrost thaw gradient. *bioRxiv*. 2025: 2025.2002.2013.637936. doi: 10.1101/2025.02.13.637936

30. Hou X, He Y, Fang P *et al.* Using artificial intelligence to document the hidden RNA virosphere. *Cell*. 2024; **187**(24): 6929-6942.e6916. doi: 10.1016/j.cell.2024.09.027

31. Favre A, Päckert M, Pauls SU *et al.* The role of the uplift of the Qinghai-Tibetan Plateau for the evolution of Tibetan biotas. *Biol Rev*. 2015; **90**(1): 236-253. doi: <https://doi.org/10.1111/brv.12107>

32. Feng X, Xing P, Tao Y *et al.* Functional traits and adaptation of lake microbiomes on the Tibetan Plateau. *Microbiome*. 2024; **12**(1): 264. doi: 10.1186/s40168-024-01979-7

33. Neri U, Wolf YI, Roux S *et al.* Expansion of the global RNA virome reveals diverse clades of bacteriophages. *Cell*. 2022; **185**: 4023-4037. doi: <https://doi.org/10.1016/j.cell.2022.08.023>

34. Wolf YI, Silas S, Wang Y *et al.* Doubling of the known set of RNA viruses by metagenomic analysis of an aquatic virome. *Nat Microbiol*. 2020; **5**(10): 1262-1270. doi: 10.1038/s41564-020-0755-4

35. Urayama S-i, Fukudome A, Hirai M *et al.* Double-stranded RNA sequencing reveals distinct riboviruses associated with thermoacidophilic bacteria from hot springs in Japan. *Nat Microbiol*. 2024; **9**(2): 514-523. doi: 10.1038/s41564-023-01579-5

36. Dominguez-Huerta G, Wainaina JM, Zayed AA *et al.* The RNA virosphere: How big and diverse is it? *Environ Microbiol*. 2023; **25**(1): 209-215. doi: <https://doi.org/10.1111/1462-2920.16312>

37. Zayed AA, Wainaina JM, Dominguez-Huerta G *et al.* Cryptic and abundant marine viruses at the evolutionary origins of Earth’s RNA virome. *Science*. 2022; **376**(6589): 156-162. doi: doi:10.1126/science.abm5847

38. Neri U, Wolf YI, Roux S *et al.* Expansion of the global RNA virome reveals diverse clades of bacteriophages. *Cell*. 2022. doi: <https://doi.org/10.1016/j.cell.2022.08.023>

39. Wu L, Liu Y, Shi W *et al.* Uncovering the hidden RNA virus diversity in Lake Nam Co: Evolutionary insights from an extreme high-altitude environment. *Proc Natl Acad Sci U S A*. 2025; **122**(6): e2420162122. doi: 10.1073/pnas.2420162122

40. Zhao Y, Zhang Z, Feng M *et al.* Functional and evolutionary characterization of potential auxiliary metabolic genes of the global RNA virome. *iMetaOmics*. 2025: e70002. doi: <https://doi.org/10.1002/imo2.70002>

41. Yuan L, Ju F. Metatranscriptomic Compendium of 55900 RNA Viruses Deciphers Human Health Implication and Ecological roles of RNA Virome in Global Wastewater Treatment Plants. *bioRxiv*. 2024: 2024.2003.2012.584551. doi: 10.1101/2024.03.12.584551

42. Tian F, Wainaina JM, Howard-Varona C *et al.* Prokaryotic-virus-encoded auxiliary metabolic genes throughout the global oceans. *Microbiome*. 2024; **12**(1): 159. doi: 10.1186/s40168-024-01876-z

43. Ju F, Beck K, Yin X *et al.* Wastewater treatment plant resistomes are shaped by bacterial composition, genetic exchange, and upregulated expression in the effluent microbiomes. *ISME J*. 2019; **13**(2): 346-360. doi: 10.1038/s41396-018-0277-8

44. Gan T, Wang D. Picobirnaviruses encode proteins that are functional bacterial lysins. *Proc Natl Acad Sci U S A*. 2023; **120**(37): e2309647120. doi: doi:10.1073/pnas.2309647120

45. Yoshida M, Medvedeva S, Fukudome A *et al.* “Paraxenoviridae”, a putative family of ubiquitous marine bacteriophages with double-stranded RNA genomes. *bioRxiv*. 2025: 2025.2002.2027.640542. doi: 10.1101/2025.02.27.640542

46. Mollentze N, Streicker DG. Predicting zoonotic potential of viruses: where are we? *Curr Opin Virol*. 2023; **61**: 101346. doi: <https://doi.org/10.1016/j.coviro.2023.101346>

47. Thompson LR, Zeng Q, Kelly L *et al.* Phage auxiliary metabolic genes and the redirection of cyanobacterial host carbon metabolism. *Proc Natl Acad Sci U S A*. 2011; **108**(39): E757-E764. doi: 10.1073/pnas.1102164108

48. Wu R, Smith CA, Buchko GW *et al.* Structural characterization of a soil viral auxiliary metabolic gene product – a functional chitosanase. *Nat Commun*. 2022; **13**(1): 5485. doi: 10.1038/s41467-022-32993-8

49. Zhang F, Shi X, Zeng C *et al.* Recent stepwise sediment flux increase with climate change in the Tuotuo River in the central Tibetan Plateau. *Sci Bull*. 2020; **65**(5): 410-418. doi: <https://doi.org/10.1016/j.scib.2019.12.017>

50. Li S, Liu Y, Wu Y *et al.* Antibiotics in global rivers. *National Science Open*. 2022; **1**(2): 20220029. doi: <https://doi.org/10.1360/nso/20220029>

51. Bolger AM, Lohse M, Usadel B. Trimmomatic: a flexible trimmer for Illumina sequence data. *Bioinform*. 2014; **30**(15): 2114-2120.

52. Kopylova E, Noé L, Touzet H. SortMeRNA: fast and accurate filtering of ribosomal RNAs in metatranscriptomic data. *Bioinform*. 2012; **28**(24): 3211-3217. doi: 10.1093/bioinformatics/bts611

53. Li D, Liu C-M, Luo R *et al.* MEGAHIT: an ultra-fast single-node solution for large and complex metagenomics assembly via succinct de Bruijn graph. *Bioinform*. 2015; **31**(10): 1674-1676.

54. Koonin EV, Dolja VV, Krupovic M *et al.* Global organization and proposed megataxonomy of the virus world. *Microbiol Mol Biol Rev*. 2020; **84**(2): e00061-00019. doi: 10.1128/mmbr.00061-19

55. Hyatt D, Chen G-L, LoCascio PF *et al.* Prodigal: prokaryotic gene recognition and translation initiation site identification. *BMC Bioinformatics*. 2010; **11**(1): 119. doi: 10.1186/1471-2105-11-119

56. Johnson LS, Eddy SR, Portugaly E. Hidden Markov model speed heuristic and iterative HMM search procedure. *BMC Bioinform*. 2010; **11**(1): 431. doi: 10.1186/1471-2105-11-431

57. Sakaguchi S, Urayama S-i, Takaki Y *et al.* NeoRdRp: A Comprehensive dataset for identifying RNA-dependent RNA polymerases of various RNA Viruses from metatranscriptomic data. *Microbes Environ*. 2022; **37**(3): ME22001. doi: 10.1264/jsme2.ME22001

58. Babaian A, Edgar R. Ribovirus classification by a polymerase barcode sequence. *PeerJ*. 2022; **10**: e14055. doi: 10.7717/peerj.14055

59. Nayfach S, Camargo AP, Schulz F *et al.* CheckV assesses the quality and completeness of metagenome-assembled viral genomes. *Nat Biotechnol*. 2021; **39**(5): 578-585. doi: 10.1038/s41587-020-00774-7

60. Wolf YI, Kazlauskas D, Iranzo J *et al.* Origins and evolution of the Global RNA Virome. *mBio*. 2018; **9**(6). doi: 10.1128/mBio.02329-18

61. Fu L, Niu B, Zhu Z *et al.* CD-HIT: accelerated for clustering the next-generation sequencing data. *Bioinform*. 2012; **28**(23): 3150-3152. doi: 10.1093/bioinformatics/bts565

62. Camargo AP, Nayfach S, Chen IMA *et al.* IMG/VR v4: an expanded database of uncultivated virus genomes within a framework of extensive functional, taxonomic, and ecological metadata. *Nucleic Acids Res*. 2023; **51**(D1): D733-D743. doi: 10.1093/nar/gkac1037

63. Edgar RC. Search and clustering orders of magnitude faster than BLAST. *Bioinform*. 2010; **26**(19): 2460-2461. doi: 10.1093/bioinformatics/btq461

64. Enright AJ, Van Dongen S, Ouzounis CA. An efficient algorithm for large-scale detection of protein families. *Nucleic Acids Res*. 2002; **30**(7): 1575-1584. doi: 10.1093/nar/30.7.1575

65. Katoh K, Misawa K, Kuma Ki *et al.* MAFFT: a novel method for rapid multiple sequence alignment based on fast Fourier transform. *Nucleic Acids Res*. 2002; **30**(14): 3059-3066. doi: 10.1093/nar/gkf436

66. Capella-Gutiérrez S, Silla-Martínez JM, Gabaldón T. trimAl: a tool for automated alignment trimming in large-scale phylogenetic analyses. *Bioinform*. 2009; **25**(15): 1972-1973. doi: 10.1093/bioinformatics/btp348

67. Letunic I, Bork P. Interactive Tree Of Life (iTOL) v5: an online tool for phylogenetic tree display and annotation. *Nucleic Acids Res*. 2021; **49**(W1): W293-W296.

68. Edgar RC, Taylor J, Lin V *et al.* Petabase-scale sequence alignment catalyses viral discovery. *Nature*. 2022; **602**: 142-147. doi: 10.1038/s41586-021-04332-2

69. Jones P, Binns D, Chang H-Y *et al.* InterProScan 5: genome-scale protein function classification. *Bioinform*. 2014; **30**(9): 1236-1240. doi: 10.1093/bioinformatics/btu031

70. Potenza E, Domenico TD, Walsh I *et al.* MobiDB 2.0: an improved database of intrinsically disordered and mobile proteins. *Nucleic Acids Res*. 2014; **43**(D1): D315-D320. doi: 10.1093/nar/gku982

71. Attwood TK, Coletta A, Muirhead G *et al.* The PRINTS database: a fine-grained protein sequence annotation and analysis resource—its status in 2012. *Database*. 2012; **2012**(bas019). doi: 10.1093/database/bas019

72. Käll L, Krogh A, Sonnhammer ELL. Advantages of combined transmembrane topology and signal peptide prediction—the Phobius web server. *Nucleic Acids Res*. 2007; **35**(suppl_2): W429-W432. doi: 10.1093/nar/gkm256

73. Krogh A, Larsson B, von Heijne G *et al.* Predicting transmembrane protein topology with a hidden markov model: application to complete genomes. *J Mol Biol*. 2001; **305**(3): 567-580. doi: <https://doi.org/10.1006/jmbi.2000.4315>

74. Steinegger M, Meier M, Mirdita M *et al.* HH-suite3 for fast remote homology detection and deep protein annotation. *BMC Bioinform*. 2019; **20**(1): 473. doi: 10.1186/s12859-019-3019-7

75. Mirdita M, von den Driesch L, Galiez C *et al.* Uniclust databases of clustered and deeply annotated protein sequences and alignments. *Nucleic Acids Res*. 2017; **45**(D1): D170-D176. doi: 10.1093/nar/gkw1081

76. Finn RD, Clements J, Eddy SR. HMMER web server: interactive sequence similarity searching. *Nucleic Acids Res*. 2011; **39**(suppl_2): W29-W37. doi: 10.1093/nar/gkr367

77. Mistry J, Chuguransky S, Williams L *et al.* Pfam: The protein families database in 2021. *Nucleic Acids Res*. 2020; **49**(D1): D412-D419. doi: 10.1093/nar/gkaa913

78. Wang J, Chitsaz F, Derbyshire MK *et al.* The conserved domain database in 2023. *Nucleic Acids Res*. 2022; **51**(D1): D384-D388. doi: 10.1093/nar/gkac1096

79. Sillitoe I, Bordin N, Dawson N *et al.* CATH: increased structural coverage of functional space. *Nucleic Acids Res*. 2020; **49**(D1): D266-D273. doi: 10.1093/nar/gkaa1079

80. Cheng H, Liao Y, Schaeffer RD *et al.* Manual classification strategies in the ECOD database. *Proteins:Struct, Funct, Bioinf*. 2015; **83**(7): 1238-1251. doi: <https://doi.org/10.1002/prot.24818>

81. Roots CT, Lukasiewicz A, Barrick JE. OSTIR: open source translation initiation rate prediction. *J Open Source Softw*. 2021; **6**(64): 3362. doi: 10.21105/joss.03362

82. Krishnamurthy SR, Wang D. Extensive conservation of prokaryotic ribosomal binding sites in known and novel picobirnaviruses. *Virology*. 2018; **516**: 108-114. doi: <https://doi.org/10.1016/j.virol.2018.01.006>

83. Yang Z, Shan Y, Liu X *et al.* VirID: Beyond Virus Discovery—An Integrated Platform for Comprehensive RNA Virus Characterization. *Mol Biol Evol*. 2024; **41**(10). doi: 10.1093/molbev/msae202

84. Yang J-H, Luo C-F, Xiang R *et al.* Host taxonomy and environment shapes insectivore viromes and viral spillover risks in Southwestern China. *Microbiome*. 2025; **13**(1): 122. doi: 10.1186/s40168-025-02115-9

85. Zhang Z, Liu Y, Zhao W *et al.* Distinct genes and microbial communities involved in nitrogen cycling between monsoon- and westerlies-dominated Tibetan glaciers. *Nat Commun*. 2025; **16**(1): 5926. doi: 10.1038/s41467-025-61002-x

86. Woodcroft BJ, Singleton CM, Boyd JA *et al.* Genome-centric view of carbon processing in thawing permafrost. *Nature*. 2018; **560**(7716): 49-54. doi: 10.1038/s41586-018-0338-1

87. Starr EP, Nuccio EE, Pett-Ridge J *et al.* Metatranscriptomic reconstruction reveals RNA viruses with the potential to shape carbon cycling in soil. *Proc Natl Acad Sci U S A*. 2019; **116**(51): 25900-25908. doi: 10.1073/pnas.1908291116

88. Roux S, Camargo AP, Coutinho FH *et al.* iPHoP: An integrated machine learning framework to maximize host prediction for metagenome-derived viruses of archaea and bacteria. *PLoS Biol*. 2023; **21**(4): e3002083. doi: 10.1371/journal.pbio.3002083

89. Hou X, He Y, Fang P *et al.* Artificial intelligence redefines RNA virus discovery. *bioRxiv*. 2023: 2023.2004.2018.537342. doi: 10.1101/2023.04.18.537342

90. Shi M, Lin X-D, Tian J-H *et al.* Redefining the invertebrate RNA virosphere. *Nature*. 2016; **540**(7634): 539-543. doi: 10.1038/nature20167

91. Chaumeil P-A, Mussig AJ, Hugenholtz P *et al.* GTDB-Tk v2: memory friendly classification with the genome taxonomy database. *Bioinform*. 2022; **38**(23): 5315-5316. doi: 10.1093/bioinformatics/btac672

92. Chklovski A, Parks DH, Woodcroft BJ *et al.* CheckM2: a rapid, scalable and accurate tool for assessing microbial genome quality using machine learning. *Nat Methods*. 2023; **20**(8): 1203-1212. doi: 10.1038/s41592-023-01940-w

93. Liu Y, Jiao N, Xu Zhong K *et al.* Diversity and function of mountain and polar supraglacial DNA viruses. *Sci Bull*. 2023; **68**(20): 2418-2433. doi: <https://doi.org/10.1016/j.scib.2023.09.007>

94. Shaffer M, Borton MA, McGivern BB *et al.* DRAM for distilling microbial metabolism to automate the curation of microbiome function. *Nucleic Acids Res*. 2020; **48**(16): 8883-8900. doi: 10.1093/nar/gkaa621

95. Mollentze N, Babayan SA, Streicker DG. Identifying and prioritizing potential human-infecting viruses from their genome sequences. *PLoS Biol*. 2021; **19**(9): e3001390. doi: 10.1371/journal.pbio.3001390

96. Sun J, Zheng K, Liang Y *et al.* Diverse, abundant and stable coastal RNA viruses identified by spatiotemporal metatranscriptomics. *Research Square*. 2022. doi: 10.21203/rs.3.rs-2282285/v1

97. Pratama AA, Bolduc B, Zayed AA *et al.* Expanding standards in viromics: in silico evaluation of dsDNA viral genome identification, classification, and auxiliary metabolic gene curation. *PeerJ*. 2021; **9**: e11447. doi: 10.7717/peerj.11447
